# Safe and Robust Map Updating for Long-Term Operations in Dynamic Environments †

**DOI:** 10.3390/s23136066

**Published:** 2023-06-30

**Authors:** Elisa Stefanini, Enrico Ciancolini, Alessandro Settimi, Lucia Pallottino

**Affiliations:** 1 Soft Robotics for Human Cooperation and Rehabilitation, Fondazione Istituto Italiano di Tecnologia, Via Morego, 30, 16163 Genova, Italy; 2 Centro di Ricerca “E. Piaggio”, Dipartimento di Ingegneria dell’Informazione, Universitá di Pisa, Largo L. Lazzarino 1, 56122 Pisa, Italy; lucia.pallottino@unipi.it; 3 Proxima Robotics s.r.l., Via Olbia, 20, 56021 Cascina, Italy; enrico.ciancolini@gmail.com (E.C.); alessandro@proximarobotics.com (A.S.)

**Keywords:** mapping, localisation, dynamic environments

## Abstract

Ensuring safe and continuous autonomous navigation in long-term mobile robot applications is still challenging. To ensure a reliable representation of the current environment without the need for periodic remapping, updating the map is recommended. However, in the case of incorrect robot pose estimation, updating the map can lead to errors that prevent the robot’s localisation and jeopardise map accuracy. In this paper, we propose a safe Lidar-based occupancy grid map-updating algorithm for dynamic environments, taking into account uncertainties in the estimation of the robot’s pose. The proposed approach allows for robust long-term operations, as it can recover the robot’s pose, even when it gets lost, to continue the map update process, providing a coherent map. Moreover, the approach is also robust to temporary changes in the map due to the presence of dynamic obstacles such as humans and other robots. Results highlighting map quality, localisation performance, and pose recovery, both in simulation and experiments, are reported.

## 1. Introduction

Autonomous mobile robots are becoming increasingly popular in industrial applications such as logistics, manufacturing, and warehousing. One of the key requirements for the operation of such robots is the ability to navigate safely and efficiently in a dynamic environment, repeatedly performing the same task all day for days or months. A mobile robot’s autonomous navigation is achieved using simultaneous localisation and mapping (SLAM) techniques, in which the robot maps the environment, navigates, and locates itself by merely ”looking” at this environment, without the need for additional hardware to be installed in the workplace. This is achieved by using sensor measurements (such as Lidar, cameras, or other sensors) to update the map and estimate the robot’s pose at the same time [1]. However, these methods face significant challenges in dynamic environments, mainly when the environment changes over time due to the movement of obstacles or people. Obstacles may appear, disappear, or move around, and the robot’s map needs to be updated continuously to reflect these changes. While localisation and local obstacle avoidance algorithms can handle small changes in the environment, the robot’s map can diverge from the present working area, reducing navigation performance [2]. Conventional SLAM methods struggle to update the map accurately and efficiently over long periods of time due to the accumulation of errors in the robot’s pose estimates and in the map. This is because small errors in the robot’s pose estimation can accumulate over time, leading to significant inaccuracies in the map. Moreover, the robot needs to constantly relocalise itself, which can be challenging and time-consuming. Indeed, traditional SLAM algorithms are computationally intensive and require significant processing power, memory, and time [3]. This can limit the robot’s ability to operate for extended periods, particularly in industrial environments where robots need to operate continuously.

Therefore, the long-term operation of autonomous mobile robots in dynamic industrial environments requires continuous map updating to ensure accurate navigation and obstacle avoidance and achieve good localisation performance [4,5]. Moreover, having an updated static map, coherent with the current environment, allows the robot to reduce its effort in replanning over time to adjust its trajectory. Updating the map in real time requires the robot to quickly detect and track changes in the environment, such as the movement of people, equipment, and obstacles. For this reason, and in order to consider long-term operations, the map-updating algorithm has to be computationally light and use limited memory [6].

In our previous paper [7], a 2D Lidar-based map-updating method to detect possible changes over time in the environment and to accordingly update a previously built map with limited memory usage while neglecting the presence of highly dynamic obstacles was proposed. The system was developed using the Robot Operating System framework [8] using a 2D occupancy grid map representation. This map representation is commonly used in the industrial field due to its ability to facilitate path-planning [9,10]. In fact, companies such as AgileX Robotics (AgileX, https://global.agilex.ai/, accessed on 28 March 2023), Robotnik, (Robotnik, https://robotnik.eu/, accessed on 28 March 2023), and Bosch (Bosch, https://www.bosch.com/, accessed on 28 March 2023) currently employ 2D occupancy grid maps in their assets, demonstrating the technology’s high level of industrial readiness. As such, it is important to continuously improve and strengthen the robustness of this technology for long-term use.

### Paper Contribution

Although the proposed map updating method [7] provides promising results in map quality and localisation performance in a dynamic environment, the method relies heavily on accurate knowledge about the initial pose of the robot in a previously built map. That information might not be available with high accuracy, and due to changes in the map, it might be hard to estimate immediately. This could lead to bias in the estimated pose of the robot, which could result in overwriting free and occupied cells, effectively corrupting the occupancy grid and the resulting map. Indeed, as the robot moves through its environment, small errors in its position estimate can accumulate, resulting in a significant deviation from its true position over time. This issue is often referred to as drift, and can be caused by a variety of factors, such as wheel slippage, sensor noise, and environmental changes.

This paper focuses on developing a map-updating algorithm that can handle localisation errors and integrate recovery procedures to ensure the map’s accuracy is not degraded during long-term operations. To address the limitations of the existing approach, a fail-safe mechanism based on localisation performance is provided. This mechanism prevents erroneous map updating when there is an increase in localisation error. Additionally, the integration of the pose update to be activated when the robot effectively gets lost is also proposed. Extensive simulations were conducted to validate the individual components of the proposed system as well as its overall performance. Furthermore, real-world experiments were conducted to demonstrate the approach’s robustness in uncertain conditions within a realistic environment.

The primary technical challenge addressed in this paper is the improvement of accuracy and robustness in map updating when localisation errors are present. This aspect is crucial for the sustained operation of autonomous mobile robots in dynamic industrial environments. Particularly, a key technical challenge is determining when the erroneous robot’s localisation may jeopardise the map reconstruction. These contributions aim to enhance the reliability, accuracy, and efficiency of map updating, addressing limitations observed in previous works. In conclusion, the proposed approach’s key strength lies in its ability to facilitate robust long-term operations.

## 2. Related Works

Over the past few decades, an immense and comprehensive body of research has emerged regarding the issue of updating maps in a dynamic environment to support long-term operations. Sometimes, researchers refer to the problem as a lifelong mapping or a lifelong SLAM one. In this section, we present the most relevant solutions proposed in the literature related to the occupancy grid map representation and methods for updating an existing one, underlining the difference with conventional SLAM methods.

### 2.1. Occupancy Grid

Occupancy grid maps are a spatial representation that captures the occupancy state of an environment by dividing it into a grid of cells. Each cell is associated with a binary random variable indicating the probability that the cell is occupied (free, occupied, or unknown). One common method for updating the occupancy grid map is to use a probabilistic model such as the Bayesian filter [11]. This filter estimates the probability distribution of the occupancy state of each cell based on the likelihood of sensor measurements and the prior occupancy state. Most occupancy grid map algorithms are derived from Bayesian filters and differ from the posterior calculation. However, the Bayesian approach has drawbacks in the difficulty of correct map updates based on possible uncertainties in the robot’s poses [12]. Although the construction of a static environment map using the occupancy grid approach has already been solved [13], its effectiveness in dynamic environments over extended periods of time still requires further investigation. Different methods have expanded the occupancy grid map algorithms to handle dynamic and semistatic environments [14,15,16,17]. These approaches assess the occupancy of cells irrespective of the type of detected obstacle, thereby mitigating the challenges related to multiobstacle detection and tracking and improving the speed of the mapping process [18]. However, their primary focus is on accelerating the mapping construction process rather than in the updating process of an initial occupancy grid map for an extended period of time. This may limit the use of such approaches in large, industrial scenarios, where a full map reconstruction requires a computational and resource effort to continuously replan the whole robot motion in the new map.

### 2.2. Lifelong Mapping

In the literature, there exist grid-based methods aimed at lifelong mapping. One group of these algorithms is based on the update of local maps, as demonstrated in [6], based on a recency-weighted averaging technique to detect persistent variations. They merge these maps using Hough transformation. However, this approach assumes that no dynamic obstacles are in the environment. In contrast, reference [19] still generates local maps and continuously updates them online, requiring a substantial memory usage. To address this, reference [20] proposed pruning redundant local maps to reduce computational costs while maintaining both efficiency and consistency. However, these systems are not currently used in industrial applications, except for closed navigation systems, and are not comparable with occupancy grid-based methods like the one proposed in this paper, because the map representation they rely on is different. Instead, in [5], the authors updated a 2D occupancy map of a nonstatic facility logistics environment using a multirobot system, where the local temporary maps built by each robot are merged into the current global one. While the approach is promising for map updating of nonstatic environments, the need for precise localisation and assumptions on the environment can be challenging in cluttered and dynamic environments, providing a limit of the method. Finally, the authors of [21], on the other hand approached each cell’s occupancy as a failure analysis problem and contributed temporal persistence modelling (TPM), which is an algorithm for probabilistic prediction of a cell’s “occupied” or “empty” state given sparse prior observations from a task-specific mobile robot. Although the introduced concept is interesting, it suffers from a limitation due to temporary map building for motion planning, and hence, it does not provide an entire updated representation of the environment.

### 2.3. Lifelong Localisation

In order to perform long-term operations in a dynamic environment, the map used by the robot to autonomously navigate needs to be up-to-date and accurately reflect the current state of the environment. To update a map correctly, a robot must first accurately localise itself within the environment. If the robot’s position is incorrect or uncertain, any updates made to the map may be inaccurate, leading to errors in navigation and task performance. In the literature, there are several works that take into account the localisation problem in order to achieve better results in the map update process, which, like our proposed approach, does not fall within SLAM systems. In [22], the author proposed a method for the long-term localisation of mobile robots in dynamic environments using time series map prediction. They produced a map of the environment and predicted the changes in the 2D occupancy grid map over time using the ARMA time series model. The robot’s localisation was then estimated by matching its sensor readings to the predicted map. The proposed method achieves better localisation accuracy and consistency compared to traditional methods that rely on static maps. However, there are some limitations related to the computational complexity of the time series model, which can make real-time implementation challenging. Additionally, the accuracy of the map prediction is highly dependent on the quality of the sensor data and the ability of the time series model to accurately capture the dynamics of the environment. In [23], Sun et al. proposed an approach for achieving the effective localisation of mobile robots in dynamic environments. They solved the localisation problem through a particle filter and scan matching. When the matching score is above a certain threshold, the map-updating step is performed. However, they considered only environments with a clearly defined static structure. In [24], instead, they discourage map updating if the robot is in an area with low localisability (i.e., the map is locally poor in environmental features). To further improve robustness, they introduced a dynamic factor to regulate how easily a map cell changes its state. Although they did not make any explicit assumption on the environment, the dynamic factor works well in scenarios where changes occur regularly (e.g., a parking lot, where the walls are fixed, and the parking spots change occupancy state frequently). Instead, it is not well-suited for environments that slowly change in an irregular and unpredictable fashion. All the works mentioned above follow the same pattern, from which we took inspiration for our approach, starting from a previously created map and applying various methods to ensure a robust localisation that leads to a correct map update. However, none of them provide results concerning the robot lost localisation case. On the contrary, our approach does not provide a continuous localisation algorithm but an evaluation of the localisation error, so as not to update the map incorrectly. Finally, our approach also handles the case of a robot getting lost in the environment due to localisation errors.

### 2.4. Conventional Lifelong SLAM

Other systems that try to solve autonomous navigation in long-term operations rely on lifelong SLAM. In such scenarios, the SLAM technique is used to provide either a new map every time the robot performs a new run or an updated map, both robust to localisation errors and environmental dynamics. Thus, they are oriented to reduce, e.g., computational performance, loop closure, and pose graph sparsification [3], resulting in a stand-alone system based on internal structure that can not be straightforwardly integrated—and hence, easily used—in industrial systems. This is also due to the fact that they may be not based on occupation grid map approaches. In [25], for example, the authors present a new technique based on a probabilistic feature persistence model to predict the state of the obstacles in the environment and update the world representation accordingly.

Due to their incremental approach, most available lifelong SLAM systems rely on graphs [26,27] or other internal structures. Even if an occupancy grid map can be built for navigation purposes, an existing graph structure is required as input rather than a previous occupancy grid map.

Finally, our intent and difference with a SLAM method should be clear. Our proposed approach is based on the localisation error evaluation of the robot’s estimated pose provided by any localisation algorithm to update the map correctly and continue its update in case of loss of the robot’s pose. The proposed approach is proven to be able to capture environmental changes while neglecting the presence of moving obstacles such as humans or other robots. Moreover, the approach is designed to guarantee limited memory usage and to be easily integrated into typical industrial mobile systems.

## 3. List of Variables

For easier reading and understanding, Table 1 reports the description of all variables used in the paper, while Table 2 reports all the parameters with their value used in the simulations/experiments. The choice of these parameters will be discussed in the paper.

## 4. Problem Formulation

Consider a mobile robot, denoted as R, equipped with a 2D Lidar and encoders, operating in a dynamic environment $W_{0}$. The robot requires a map and a localisation algorithm to autonomously navigate. The latter provides a global estimated robot’s pose ${\tilde{X}}_{R}\left(k\right)=\left[\begin{array}{c} \tilde{p}\left(k\right)\;\tilde{\theta }\left(k\right) \end{array}\right]$, with $\tilde{p}\in R^{2}$ and $\tilde{\theta }\in R$ being the robot estimated position and orientation, respectively, while the actual pose is represented by $X_{R}\left(k\right)=\left[\begin{array}{c} p(k)\;\theta (k) \end{array}\right]$. The goal is to keep the localisation error $e_{l}\left(k\right)$, defined as the difference between the estimated and actual poses, below a threshold ϵ to allow the robot to successfully complete its task.

In dynamic environments, the arrangement of obstacles changes over time, creating new environments $W_{i}$ that are similar to the previous ones $W_{i-1}$. However, using an initial static map $M_{0}$ leads to localisation errors when the map no longer reflects the current configuration of $W_{i}$. To address this, a solution is to update the map, providing a new $M_{i}\left(k\right)$, ensuring that $e_{l}\left(k\right)<\epsilon$.

**Figure 1:**
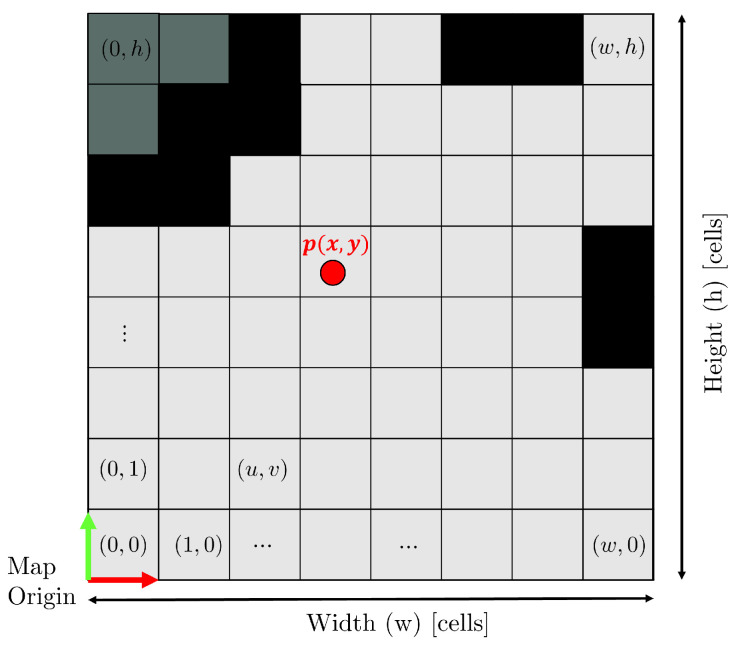
Two-dimensional occupancy grid map representation: The origin of grid coordinates is in the bottom-left corner, with the first location having an index of (0,0).

Occupancy Grid Map: The paper employs the occupancy grids technique to represent and model the environment. A 2D occupancy grid map example is depicted in Figure 1. It is a 8x8-cell grid-based representation of the robot’s surrounding space. Each cell in the grid, denoted as $c_{j}\left(q\right)$, provides information about the area associated with a position $q\in R^{2}$ in the space. As we can see in Figure 1, the probability assigned to each cell indicates whether it is occupied (probability 1, black cells), free (probability 0, grey cells), or unknown (dark grey cells). The cells are identified using grid coordinates (u,v), which define the resolution *r* of the occupancy grid and the locations of obstacles.

Various sensors can be employed to build 2D occupancy grid maps, including Lidars, cameras, sonar, and infrared sensors. However, this paper specifically focuses on using 2D Lidars to generate a 2D point cloud. It is worth noting that the proposed method is applicable to all sensors able to generate point clouds.

Lidar Point Cloud: Given a laser range measurement $Z\left(k\right)={\left[\begin{array}{c} z_{1}\left(k\right)\ldots z_{n}\left(k\right) \end{array}\right]}^{T}$ with *n* laser beams at time *k*, it is possible to transform the data in a point cloud $P\left(k\right)={\left[\begin{array}{c} p_{1}\left(k\right)\ldots p_{n}\left(k\right) \end{array}\right]}^{T}$, where $p_{i}\left(k\right)\in R^{2}$ is the *i*-th hit point at time step *k*, i.e., the point in which the *i*-th beam hits an obstacle. Moreover, given Z(k) and the estimated robot’s pose ${\tilde{X}}_{R}\left(k\right)$, it is possible to obtain the coordinates of $p_{i}\left(k\right)$ in a fixed frame as to ease the notation, the time dependence *k* is omitted):

(1) $$\begin{array}{c} p_{i}=\left(\begin{array}{c} x_{i}\\ y_{i} \end{array}\right)=\tilde{p}+z_{i}\left(\begin{array}{c} \cos(\theta_{0}+i\;\Delta \theta +\tilde{\theta })\\ \sin(\theta_{0}+i\;\Delta \theta +\tilde{\theta }) \end{array}\right), \end{array}$$

where $x_{i},\;y_{i}\in R$ are the Cartesian coordinates of the hit point $p_{i}$ along the *i*-th laser range measurement $z_{i}$. The angular offset of the first laser beam with respect to the orientation of the laser scanner is represented by $\theta_{0}\in R$, considering that the angular rate between adjacent beams is Δθ∈R. Finally, given $p_{i}$, the set of cells passed through by the measurement is $C_{p_{i}}=\left\{\begin{array}{c} c_{1},\ldots ,c_{\mu } \end{array}\right\}$, where $c_{\mu }=c\left(p_{i}\right)$ is the cell associated to $p_{i}$.

### Ideal Scenario vs. Real Scenario

**Figure 2:**
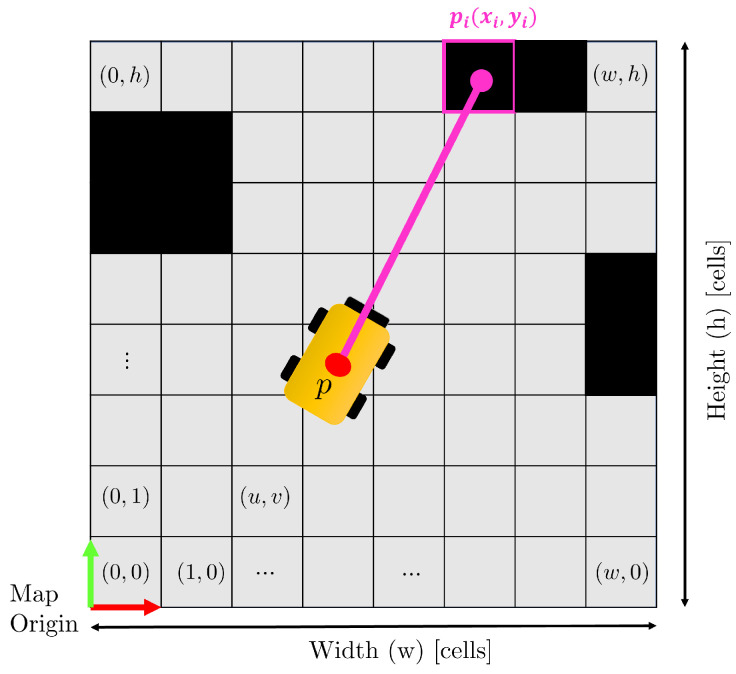
Ideal case: the laser beam hits an obstacle in $p_{i}$. The point identifies the right occupied cell in the map thanks to a correct estimation of the robot position p.

In an ideal scenario, a mobile robot equipped with a 2D Lidar operates in an environment represented by a 2D occupancy grid map. In this ideal case (Figure 2), the laser beam accurately hits an obstacle in a single occupied cell, and the robot’s pose estimation is precise.

**Figure 3:**
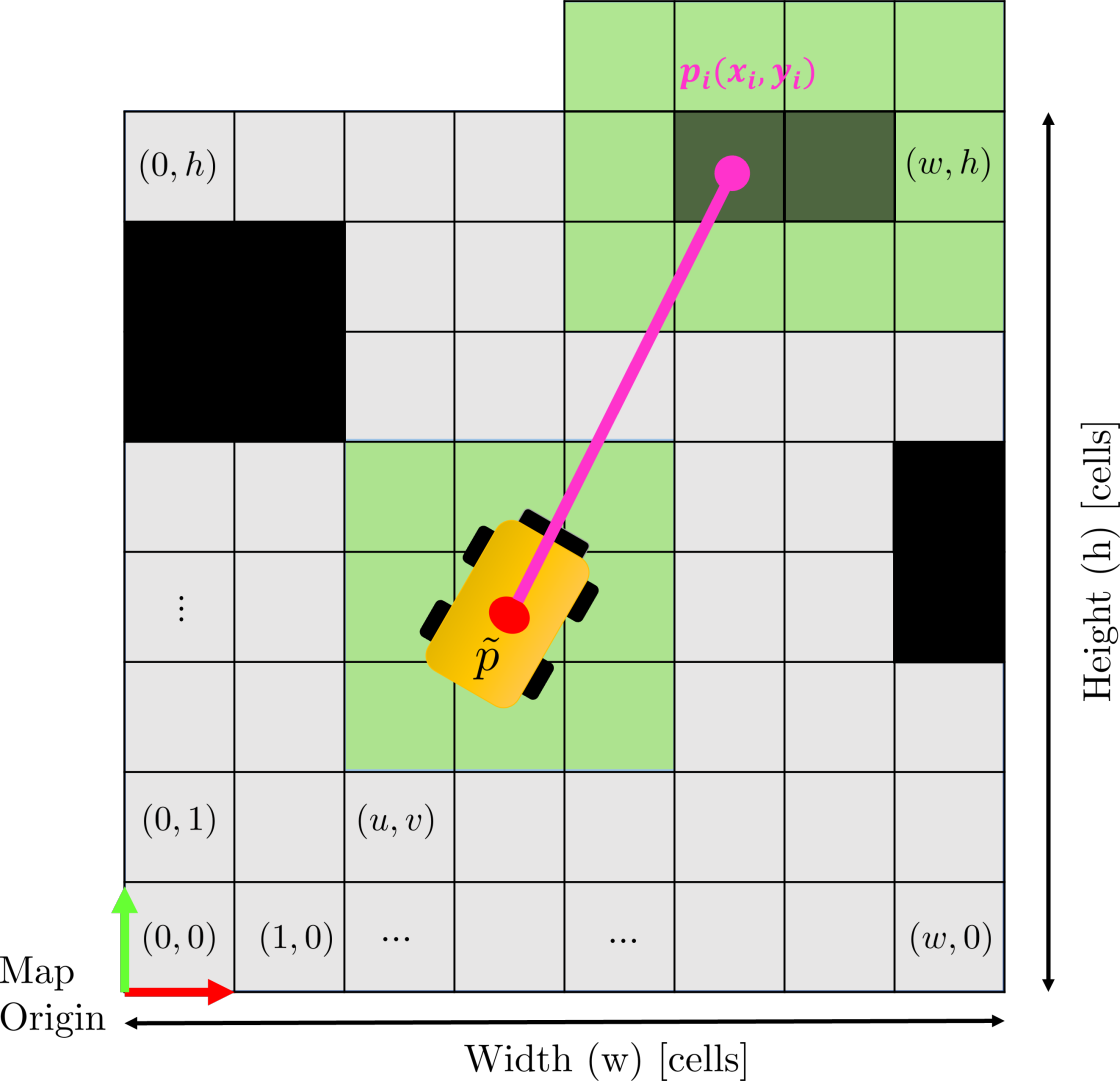
Real case: due to localisation errors on the robot pose and measurement errors or noises, the laser beams can be prevented to identify the right cell associated with the hit obstacle but another neighbouring cell.

However, in reality (Figure 3), localisation errors introduce uncertainty in the robot’s pose, represented by the green cells surrounding the robot. The combination of localisation errors, measurement errors, and noise can cause the laser beam to identify a neighbouring cell (green cells around $p_{i}$) instead of the correct one. Consequently, directly relying on the identified cell’s occupancy may introduce errors during the map-updating process. Therefore, it is crucial to develop a robust procedure that can handle these localisation and measurement errors and ensure accurate map updates.

## 5. Method

**Figure 4:**
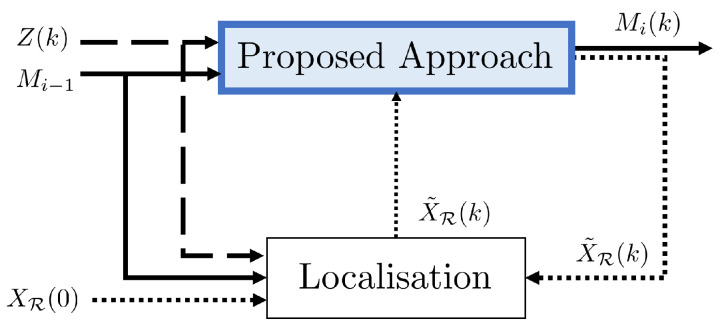
System overview: our proposed approach takes as inputs an initial occupancy grid map $M_{i-1}$, the robot pose ${\tilde{X}}_{R}\left(k\right)$ provided by a localisation algorithm, and the current laser measurements Z(k) to compute a newly updated map $M_{i}$ and a new robot pose ${\tilde{X}}_{R}\left(k\right)$ according to the localisation error.

The system architecture, depicted in Figure 4, is based on an initial occupancy grid map $M_{i-1}\left(k\right)$, robot pose ${\tilde{X}}_{R}\left(k\right)$, and current laser measurements Z(k), to compute a newly updated map $M_{i}$ and a new robot pose ${\tilde{X}}_{R}\left(k\right)$ accordingly to the localisation error.

The system algorithm, as described in Algorithm 1, begins by setting as occupied all unknown cells during the measurement process to simplify detection (line 2). Then, it classifies laser measurements as either “detected change measurement” (DCM) or “non-detected change measurement” (Non-DCM) using the *beams classifier*, (line 3), by checking for discrepancy of the measurements with the initial map $M_{i-1}$ as described in Section 5.1 and by Algorithm 2. Detected change measurements are evaluated by the *localisation check*, as described in Section 5.2, to assess the localisation error (line 5). If the number of detected changes, $n_{dc}$, is below a threshold $n_{min}$ as defined in Section 5, the *map-updating process* continues (line 5).

**Algorithm 1** Safe and robust map updating.1:**function** $M_{i}=$Safe_and_Robust_Map_Updating($Z\left(k\right),M_{i-1},{\tilde{X}}_{R}\left(k\right)$)2:    $Change\_unknown\_cells\_state\_to\_occupied(M_{i-1})$3:    [DCM, Non-DCM] = Beam_Classifier(Z(k), $M_{i-1}$,${\tilde{X}}_{R}\left(k\right)$)4:    **if** ($n_{dc}\leq n_{min}$) **then**                              ▷ (5)5:        $M_{i}=Map\_Updating\left(DCM,Non-DCM,M_{i-1},{\tilde{X}}_{R}\left(k\right)\right)$6:    **else**                                     ▷ (6)7:        $[M_{i},{\tilde{X}}_{R}\left(k\right)]=Stop\_Map\_Updating\left(\right)$8:        **if** ($n_{dc}\geq n_{max})$**then**                             ▷ (7)9:           $Pose\_Updating(Z\left(k\right),M_{i}\left(k\right),{\tilde{X}}_{R}\left(k\right))$10:        **end if**11:    **end if**12:    **Return** $M_{i}$13:**end function**

**Algorithm 2** Beam classifier function.
1:**function** [DCM, Non-DCM] =Beam_Classifier($Z\left(k\right),M_{i-1},{\tilde{X}}_{R}\left(k\right)$)2:    P(k)=Compute_hit_Point_cloud(Z(k))3:    $Z_{e}=Compute\_Z_{e}\left(M_{i-1},{\tilde{X}}_{R}\left(k\right)\right)$4:    **for each** $z_{e,i}\in Ze$ **do**5:        $p_{e,i}=Compute\_p_{e,i}\left(z_{e,i},{\tilde{X}}_{R}\left(k\right)\right)$                                               ▷ (2)6:        $Compute\_P_{e,i}\left(p_{e,i},v,n_{e}\right)$                                                   ▷ (3)7:        **for each** $p_{i}\in P\left(k\right)$ **do**8:           **if** $\min_{p_{e,i}\;\in \;P_{e,i}}{∥p_{i}-p_{e,i}∥}_{2}>D_{t}h.$ **then**                                            ▷ (4)9:               $DCM.append(p_{i})$10:           **else**11:               $Non-DCM.append(p_{i})$12:           **end if**13:        **end for**14:    **end for**15:    **Return** [DCM, Non-DCM]16:
**end function**



The map-updating process, described in Algorithm 3, detects changes in static obstacles. If the localisation check is successful, the *changed cells evaluator* and the *unchanged cells evaluator* compare current measurements $z_{i}\left(k\right)$ with the initial map $M_{i-1}\left(k\right)$ to confirm the detection of changes (lines 2–3). Such confirmation procedures, described by Algorithms 4 and 5, are used to address localisation errors and measurement noises.

A rolling buffer $B_{c_{j}}$ of fixed size $N_{b}$ is created for each cell $c_{j}\in C_{pi}$ to record evaluator outcomes at different time instants. The *changed cells evaluator* fills the buffer with a “changed” flag if the change in the measurement is detected and also confirmed as described in Section 5.3. On the other hand, the unchanged cells evaluator fills the buffer with an “unchanged” flag if the change is correctly not nondetected, as described in Section 5.4. When the robot has substantially changed its position and/or orientation, the *cell update* module changes the state of evaluated cells creating the new map $M_{i}$, as described in Section 5.5 and by Algorithm 6. This module is the one responsible for the detection of changes in the environment while neglecting dynamic obstacles.

Finally, if the number of detected changes, $n_{dc}$ evaluated by the localisation check, is above a threshold $n_{min}$, the map-updating process is suspended until the localisation algorithm recovers the right estimated pose. Moreover, if the number of detected changes exceeds $n_{max}$ as defined in Equation (7), the *pose-updating process* is activated to estimate a new robot pose (line 9) based on last $M_{i}\left(k\right)$, ${\tilde{X}}_{R}\left(k\right)$ (line 7) and Z(k) as described in Section 5.6 and by Algorithm 7.
**Algorithm 3** Map-updating function1:**function** $M_{i}=$Map_Updating(DCM, Non-DCM,$M_{i-1}$,${\tilde{X}}_{R}\left(k\right)$))2:    $Changed\_Cells\_Evaluator(DCM,M_{i-1},{\tilde{X}}_{R}\left(k\right))$3:    $Unchanged\_Cells\_Evaluator(Non-DCM,M_{i-1},{\tilde{X}}_{R}\left(k\right)))$4:    **if** Robot position displacement > $\epsilon_{p}$ || Robot orientation displacement > $\epsilon_{\theta }$ **then**5:        $M_{i}=Cells\_Update\left(\right)$6:    **end if**7:    **Return** $M_{i}$8:**end function**

### 5.1. Beam Classifier

To detect a change in the environment, the outcomes of measurement $z_{i}\left(k\right)$ must be compared to the ones the robot would obtain if it were in an environment fully corresponding to what memorized in map $M_{i-1}\left(k\right)$. The correct hit point as in (1), computed at line 2 in Algorithm 2, is hence compared to the one computed with $z_{e,i}\left(k\right)\in Z_{e}\left(k\right)$, (line 3 in Algorithm 2), which is the expected value for the *i*-th range measurement $z_{i}\left(k\right)$ obtained from the initial map $M_{i-1}$. A ray-casting approach [28], is hence used to compute the corresponding expected hit point $p_{e,i}\left(k\right)\in R^{2}$ (line 5 in Algorithm 2):

(2) $$\begin{array}{c} p_{e,i}=\left(\begin{array}{c} x_{e,i}\\ y_{e,i} \end{array}\right)=\tilde{p}+z_{e,i}\left(\begin{array}{c} \cos(\theta_{0}+i\;\Delta \theta +\tilde{\theta })\\ \sin(\theta_{0}+i\;\Delta \theta +\tilde{\theta }) \end{array}\right). \end{array}$$

To detect a change, there is not a direct comparison between the measured hit point $p_{i}\left(k\right)$ and the expected hit one $p_{e,i}\left(k\right)$. Indeed, a 1-to-N comparison strategy is applied between $p_{i}\left(k\right)$ and a set of expected hit points $P_{e,i}\left(k\right)$ consisting of hit points along virtual neighbouring range measurements.

Note that (2) computes the expected hit point $p_{e,i}$ as the sum of an offset $\tilde{p}$ and a point with polar coordinates $\left(z_{e,i},\theta_{0}+i\;\Delta \theta +\tilde{\theta }\right)$, the set $P_{e,i}$ is generated by adding different perturbations *v* to the angular second point. In more detail, the set $P_{e,i}=\left\{p_{e,i}^{\left(-n_{e}\right)},\;\ldots ,\;p_{e,i}^{\left(0\right)},\;\ldots ,\;p_{e,i}^{\left(n_{e}\right)}\right\}$ of $N=2n_{e}+1$ points is generated by adding a perturbation *v* to the angular components of $p_{e,i}$ in (2), (line 6 in Algorithm 2):

(3) $$\begin{array}{c} \begin{array}{cc} \; & p_{e,i}^{\left(l\right)}=\tilde{p}+z_{e,i}^{\left(lv\right)}\left(\begin{array}{c} \cos(\theta_{0}+\left(i+lv\right)\;\Delta \theta +\tilde{\theta })\\ \sin(\theta_{0}+\left(i+lv\right)\;\Delta \theta +\tilde{\theta }) \end{array}\right), \end{array} \end{array}$$

where *l* is the integer and $l=-n_{e},\;\ldots ,\;0,\;\ldots ,\;n_{e}$, while $|v|<\frac{\Delta \theta }{n_{e}}$ and $n_{e}\in N_{0}$ are design parameters. The distance $z_{e,i}^{\left(lv\right)}\in R^{+}$ is defined, in an analogous way to $z_{e,i}$, as the measurement in $M_{i-1}$ along the virtual laser ray of amplitude (i+lv)Δθ with respect to the *i*-th real ray. Note that *v* is chosen so that perturbations $\left(i+lv\right)\;\Delta \theta \in \left((i-1)\;\Delta \theta ,\;(i+1)\;\Delta \theta \right)$, and hence is between the i−1-th and the i+1-th laser rays.

**Figure 5:**
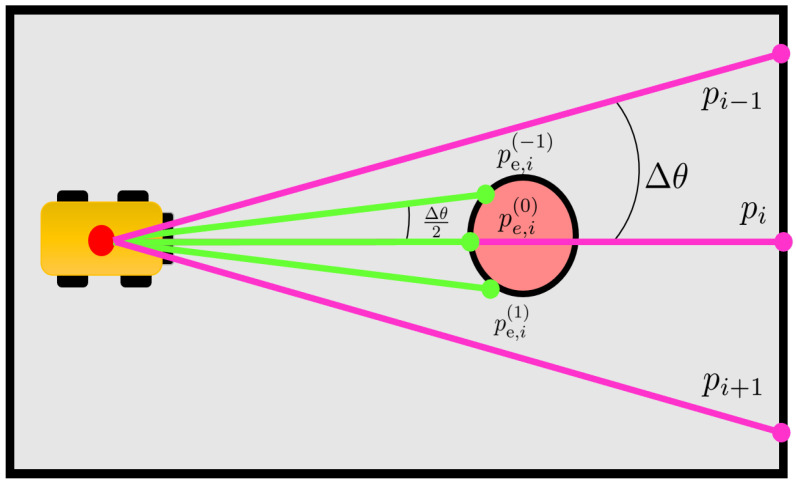
Example of $P_{e,i}=\left\{p_{e,i}^{\left(-1\right)}\;p_{e,i}^{\left(0\right)},\;p_{e,i}^{\left(1\right)}\right\}$ (in green) with N=3, $n_{e}=1$, and $v=\frac{\Delta \theta }{2}$ associated with the measured hit point $p_{i}$ (pink dot). *v* is chosen so that perturbations $\left(i+lv\right)\;\Delta \theta \in \left((i-1)\;\Delta \theta ,\;(i+1)\;\Delta \theta \right)$, and hence, between $p_{i-1}$ and $p_{i+1}$. In this map, the red obstacle has been removed with respect to $M_{i-1}$.

An example of $v=\frac{\Delta \theta }{2}$, $n_{e}=1$, and hence, N=3, is reported in Figure 5. These values are the same used for the experiments as reported in Table 2. Their choice was a trade-off between mitigating localization and noise errors and the computational cost of the procedure for creating the set $P_{e,i}$.

For the 1-to-N comparison, each point $p_{i}$ is compared to the entire set $P_{e,i}$ by computing the minimum Euclidean distance between $p_{i}$ and the *N* points in $P_{e,i}$. Finally, a change is detected only if the minimum distance between $p_{i}$ and the expected points in $P_{e,i}$ is greater than a threshold $D_{t}h$ (line 8 in Algorithm 2):

(4) $$\min_{p_{e,i}\;\in \;P_{e,i}}{∥p_{i}-p_{e,i}∥}_{2}>D_{\mathrm{th}}.$$

As described in detail in Appendix A.1, the threshold can depend linearly on the distance $z_{i}$ in order to take into account errors coming from localisation and measurement noises.

**Figure 6:**
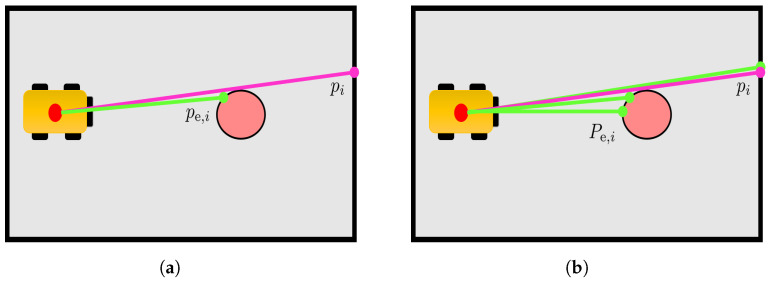
Example of a measured hit point $p_{i}$ (pink dot) and differences between single hit point and set of expected measurements (in green) in presence of an obstacle removed from previous map $M_{i-1}$. (**a**) Estimated hit point $p_{e,i}$; (**b**) set of expected measurements $P_{e,i}$ with $n_{e}=1$.

The motivation for the 1-to-N comparison is represented in Figure 6: the removal of an obstacle (in red) creates a change in a portion of the map, and a false change detection associated with the hit point computation may occur. Figure 6a shows the hit point $p_{i}$ identifying a cell of the new map that has not changed compared to the previous map $M_{i}$. On the other hand, small localisation errors may lead to an associated $p_{e,i}$ identifying an occupied cell belonging to the obstacle in $M_{i-1}$. A direct comparison between $p_{i}$ and $p_{e,i}$ creates an incorrect change detection. This does not happen in Figure 6b, where thanks to the 1-to-N comparison with the set $P_{e,i}$ the presence of a $p_{e,i}^{\left(l\right)}$ close to $p_{i}$ prevents the incorrect change detection.

### 5.2. Localisation Check

Changes in the map may have two drawbacks: first, they may prevent the robot from correctly localising itself in the changed map; second, as a consequence, large localisation errors may lead to an incorrect update of the map. Hence, monitoring the size of the localisation error is fundamental for a correct map update. For this purpose, the proposed dedicated *localisation check* module takes in input all the “detected change” measurements from the *beam classifier* and provides a quantification of the current localisation error. This module is important to make the entire system more robust by preventing corrupted map updates and by triggering a pose-updating algorithm to decrease the localisation error. The idea is to evaluate the size of the localisation error, and until this is below a given threshold, the map can be updated, neglecting the error. On the other hand, if it is too large, the robot is considered to be definitely lost, and a pose update is requested to a dedicated algorithm. On the other hand, in between those two extreme cases and thresholds, it is possible to have a sufficiently large localisation error that would provide a corrupted map update, but not so large to consider the robot definitely lost. In this case, the map update is interrupted and restarted once the localisation error decreases below the corresponding threshold.

More formally, given the number *n* of hit points in P(k), derived from laser measurement Z(k), and the number $n_{dc}\leq n$ of the “detected change” measurements, the map is updated as described next if

(5) $$n_{dc}\leq n_{min},\;n_{max}=D_{\epsilon_{min}}\cdot n$$

while the robot is considered lost, but with the possibility of recovering the localisation and the map is not updated, if the following holds:

(6) $$n_{min}<n_{dc}<n_{max}$$

Finally, the localisation system is considered not able to recover the pose, and this must be updated by a dedicated algorithm, if

(7) $$n_{dc}\geq n_{min},\;n_{max}=D_{\epsilon_{max}}\cdot n$$

where $D_{\epsilon_{min}}\in \left[0,1\right]$ is the minimum fraction of acceptable changed measurements with respect to the number of laser beams that allows for a good localisation performance, while $D_{\epsilon_{max}}\in \left[0,1\right]$ is the maximum fraction of acceptable changed measurements with respect to the number of laser beams, in addition to which the robot has definitively lost its localisation. The critical parameter to choose is $D_{\epsilon_{min}}$, because it is a trade-off between the great change between the previous and current environments and the likelihood that you have actually lost your localisation. In this paper, the values $D_{\epsilon_{min}}=0.75$ and $D_{\epsilon_{max}}=0.9$, as reported in Table 2, are considered based on the results of several experimental tests.

### 5.3. Changed Cells Evaluator

**Figure 7:**
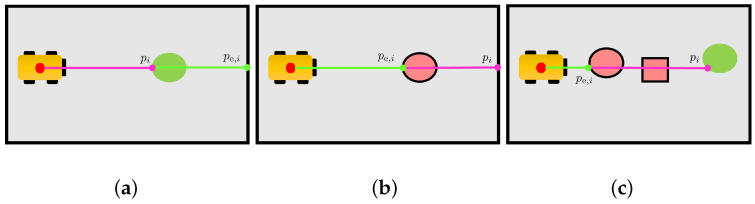
Examples of “detected change” measurements caused by an environmental change, such as the addition or removal of obstacles. Pink lines represent laser beams, while pink dots represent the measured hit points. The expected hit points computed on $M_{i}-1$ are highlighted in green. (**a**) A new obstacle has been added to the environment; (**b**) an obstacle has been removed from the environment; (**c**) two removed obstacles and a new added one.

Let $state\left(c_{j}\right)\in \left\{free,occupied\right\}$ represent the occupancy state of a cell $c_{j}$ in the initial map $M_{i-1}$. Once a change is detected by the *beam classifier* along the *i*-th range measurement $z_{i}$, it is necessary to evaluate whose states of cells in $C_{p_{i}}=\left\{\begin{array}{c} c_{1},\ldots ,c_{n} \end{array}\right\}$ must be modified, i.e., the cells passed through by *i*-th laser ray (line 3 in Algorithm 4). The *changed cells evaluator* is the module that determines which are the cells in $c_{j}\in C_{p_{i}}$ involved in the detected change. Once a change is confirmed in a cell, the module stores a “changed” flag in the buffer of each cell. The state of a cell will be changed based on how many “changed” flags are in its buffer at the end of the procedure, as described in Section 5.5. The two possible events that cause a change in the environment are illustrated in Figure 7: the appearance of a new obstacle (Figure 7a) and the removal of an existing one (Figure 7b). A combination of these two events may also occur (Figure 7c). The following paragraphs will describe how the cells in $C_{p_{i}}$, i.e., along the beam, are checked by the *changed cells evaluator* module in the occurrence of such events. A distinction between the cell $c(p_{i})$ and all other cells along the ray, i.e., $c_{j}\in C_{p_{i}}\setminus c(p_{i})$, is necessary for the evaluation, as described next.
**Algorithm 4** Changed cells evaluator function.1:**function** Changed_Cells_Evaluator(DCM)2:    **for each** $p_{i}\in$ DCM **do**3:        $C_{p_{i}}=Compute\_C_{p_{i}}\left(p_{i}\right)$4:        **for each** $c_{j}\in C_{p_{i}}$**do**                                       ▷ Change detection of cells in $C_{p_{i}}p_{i})$5:           **if** $∥p_{t}h-p_{i}{∥}_{2}<{∥p_{c_{j}}-p_{i}∥}_{2}$**then**                                                  ▷ (8)6:               **if** $state(c_{j})$ == occupied **then**7:                   $B_{c_{j}}.append\left(‘‘changed^{\prime \prime }\right)$8:               **end if**9:           **end if**10:        **end for**11:        **for each** q∈ neighbouring of $p_{i}$ **do**                                   ▷ Change detection of $c(p_{i})$, (9)12:           **if** state(c(q)) == free **then**13:               $B_{c(p_{i})}.append\left(‘‘changed^{\prime \prime }\right)$14:           **end if**15:        **end for**16:    **end for**17:**end function**

#### 5.3.1. Change Detection of Cells in $C_{p_{i}}\setminus c(p_{i})$

**Figure 8:**
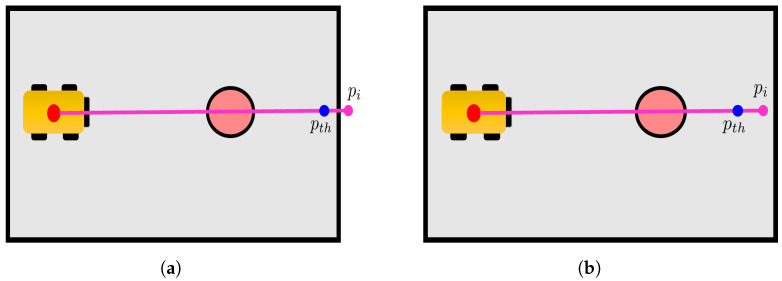
Example of localisation and noise errors leading to incorrect detection of hit point $p_{i}$ (in pink); for this reason, only cells up to $p_{t}h$ (blue) are examined for change detection. (**a**) Localisation and noise errors lead to a hit point $p_{i}$ beyond the obstacle, (**b**) Localisation and noise errors lead to a hit point $p_{i}$ in front of the obstacle.

A first evaluation is conducted on the cells along the ray, except for $c(p_{i})$, which corresponds to the hit point $p_{i}$ (lines 4–10 in Algorithm 4). Indeed, all those cells should have a *free* state, since they correspond to points of the laser beam that are not hit points. To increase algorithm robustness, we choose to not modify the state of the cells corresponding to the laser beam final chunk, since such cells may correspond to points that actually lie on obstacles due to localisation errors. For this reason, only cells between the robot’s estimated position $\tilde{p}$ and a point $p_{t}h$ that lie on the laser beam (i.e., on the segment with extremes $\tilde{p}$ and $p_{i}$) are evaluated, see Figure 8. More formally, given a fixed parameter m∈[0,1] and $p_{t}h=m\tilde{p}+\left(1-m\right)p_{i}$, only the cells $c_{j}\in C_{p_{i}}$ that satisfy (8) (line 5 in Algorithm 4) are analysed:

(8) $$∥p_{\mathrm{th}}-p_{i}{∥}_{2}<∥p_{c_{j}}-p_{i}{∥}_{2},$$

where $p_{c_{j}}\in R^{2}$ is the point in the centre of the cell $c_{j}$. If a cell $c_{j}$ satisfies (8) with $state(c_{j})=\mathrm{occupied}$ (line 6 in Algorithm 4) it means that an obstacle has been removed with respect to $M_{i-1}$, and a “changed” flag is hence added to the cell’s buffer to report this change (line 7 in Algorithm 4).

#### 5.3.2. Change Detection of $c(p_{i})$

The state of $c(p_{i})$ must also be evaluated (lines 10–15 in Algorithm 4). In an ideal situation, when a new obstacle is added, the corresponding hit point, $p_{i}$, lies on the new obstacle. In this case, the associated cell $c(p_{i})$ should have a “free” state in the previous map $M_{i-1}$ and needs to be changed to an “occupied” state. Conversely, when an obstacle is removed, the hit point $p_{i}$ lies on another obstacle or part of the environment. Therefore, the associated cell $c(p_{i})$ should have an “occupied” state in $M_{i-1}$ and should not be changed. However, due to localization errors, the distinction between added and removed obstacles becomes challenging. The cell $c(p_{i})$ corresponding to the measured hit point may be erroneously classified as “free” in both cases. To overcome this issue, the evaluation of the state of neighbouring cells is necessary. In the case of an added obstacle, all cells sufficiently close to $c(p_{i})$ should be “free” in $M_{i-1}$. On the other hand, in the case of a removed obstacle, some of these neighbouring cells would be “occupied”. By checking the states of these neighbouring cells, it hence becomes possible to distinguish between added and removed obstacles, even in the presence of localization errors. For a more detailed explanation, see Appendix A.2. To conclude, a new “changed” flag is effectively added to the buffer of $c(p_{i})$ if the added obstacle is detected, i.e., the state of neighbouring cells is *free* (line 12 in Algorithm 4); more formally, if

(9) $$\begin{array}{c} \mathrm{state}\left(c\left(q\right)\right)=\mathrm{free},\;\forall q\in R^{2}|{∥q-p_{i}∥}_{2}<tol\left(z_{i}\right), \end{array}$$

where $q\in R^{2}$ is a point in the space and $tol\in R^{+}$ is a function of the measured range as $D_{t}h$ in (4).

### 5.4. Unchanged Cells Evaluator

In case the *beam Classifier* does not detect changes along the *i*-th range measurement $z_{i}$, none of the map cells $c_{j}\in C_{p_{i}}$ associated with $z_{i}$ undergo any changes with respect to the initial map $M_{i-1}$. To take into account localisation errors, a similar procedure to the one described in the previous section can be applied, and only cells between the robot’s estimated position $\tilde{p}$ and the point $p_{th}$ that lie on the laser beam are evaluated. In particular, for such cells, the *unchanged cells evaluator* adds an “unchanged” flag to their buffer (lines 4–8 in Algorithm 5). Also, for cell $c(p_{i})$, localisation errors must be taken into account. Indeed, since the *beam classifier* does not detect any change, the state of cell $c(p_{i})$ should be *occupied* in map $M_{i-1}$, but localisation and noise errors may lead to a measured hit point $p_{i}$ with the associated *free* cell and nearby *occupied* cells. Therefore, if $state\left(c(p_{i})\right)=free$, an “unchanged” flag is added to the buffers of all *occupied* cells adjacent to $c(p_{i})$ (lines 9–12 in Algorithm 5). Otherwise, an “unchanged” flag is added only to the buffer of $c(p_{i})$ (lines 14 in Algorithm 5).
**Algorithm 5** Unchanged cells evaluator function.1:**function** Unhanged_Cells_Evaluator(Non-DCM)2:    **for each** $p_{i}\in$ Non-DCM **do**3:        $C_{p_{i}}=Compute\_C_{p_{i}}\left(p_{i}\right)$4:        **for each** $c_{j}\in C_{p_{i}}$**do**                                     ▷ Unchange detection of cells in $C_{p_{i}}p_{i})$5:           **if** $∥p_{t}h-p_{i}{∥}_{2}<{∥p_{c_{j}}-p_{i}∥}_{2}$**then**                                               ▷ (8)6:               $B_{c_{j}}.append\left(‘‘unchanged^{\prime \prime }\right)$7:           **end if**8:        **end for**9:        **if** $state(c\left(p_{i}\right))$ == free **then**10:           **for each** *q* adjacent to $p_{i}$ **do**                                     ▷ Unchange detection of $c(p_{i})$11:               $B_{c(q)}.append\left(‘‘unchanged^{\prime \prime }\right)$12:           **end for**13:        **else**14:           $B_{c(p_{i})}.append\left(‘‘unchanged^{\prime \prime }\right)$15:        **end if**16:    **end for**17:**end function**

### 5.5. Cells Update

Once each ray $z_{i}$ has been processed by the *beam classifier* and cells along the ray have been evaluated by the two previously described modules, the *cells updating* module is responsible for updating/changing the state of each cell $c_{j}$ in $C_{p_{i}}$ for all measurements $z_{i}$. This update occurs at a rate determined by linear and angular displacement thresholds, namely $\epsilon_{p}=0.05\left[m\right]$ and $\epsilon_{\theta }=20^{\circ }$ (Table 2). These parameters’ values are chosen based on the size of the environment, where larger environments correspond to lower thresholds. For each cell $c_{j}$, this update is based on the occurrence of “changed” flags in the corresponding buffer $B_{c_{j}}$. Let $N_{b}$ denote the size of the rolling buffers, and let $n_{c_{j}}\in \left\{0,\ldots ,N_{b}\right\}$ count the occurrences of the “changed” flag in $B_{c_{j}}$, computed at line 4 in Algorithm 6. A cell’s occupancy state is changed only if $n_{c_{j}}$ exceeds a given threshold $\epsilon_{n_{c_{j}}}$ (line 5 in Algorithm 6), which balances the likelihood of false positives with the speed of map updates and change detection. This threshold, and $N_{b}$ itself, are critical parameters; the threshold is used to make the updated cell state more robust with respect to dynamic events in the environment, while $N_{b}$ represents the memory of past measurements. Indeed, with a good balance in the choice of such parameters, using this approach, highly dynamic obstacles are initially detected as changes in measurements but discarded during cell update if there are an insufficient number of “changed” flags in the corresponding buffer, and hence are not considered in the new map $M_{i}$. In the experiments, we chose $N_{b}=10$, $\epsilon_{n_{c_{j}}}=7$ (Table 2), after a trial-and-error procedure.

**Figure 9:**
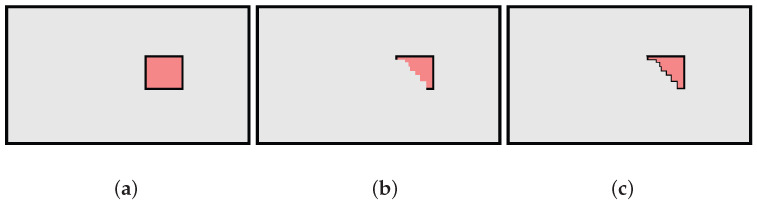
Last check of *free* cells to reconstruct obstacle borders. (**a**) Obstacle is removed from the environment; (**b**) part of the obstacle is removed from the map resulting in new *free* cells; (**c**) the unknown border cells are marked as *occupied* during the last check in order to rebuild the edge.

It is worth noting that a change in cell state from *occupied* to *free* can lead to a partial removal of an obstacle. Referring to Figure 9, cells corresponding to the border of an obstacle can hence be set as *free*, leading to an updated map $M_{i}$ with obstacles without borders characterized by measurable cells with *unknown* state; see Figure 9b, where black cells are *occupied* while red ones are *unknown*. To avoid this, the state of those cells must be set to *occupied*, leading to the creation of a new border, as in Figure 9c, lines 11–13 in Algorithm 6. At this point, the cell state update procedure is finished, and the new map $M_{i}$ can be created. To carry this out, it is important to recall that cells in the initial map $M_{i-1}$ with an “unknown” state have been considered, and treated, as *occupied* cells in the measurement process (Algorithm 1). However, if their state has not been changed to *occupied* to recreate an obstacle border, they are still represented in $M_{i}$ as “unknown” cells (lines 9–10 in Algorithm 6).
**Algorithm 6** Cells update function.1:**function** Cells_Update()
2:    **for each** $C_{p_{i}}$ **do**3:        **for each** $c_{j}\in C_{p_{i}}$ **do**4:           $n_{c_{j}}=Compute\_n_{c_{j}}\left(B_{c_{j}}\right)$5:           **if** $n_{c_{j}}>\epsilon_{n_{c_{j}}}$ **then**6:               $M_{i}\left(c_{j}\right)=1-M_{i}\left(c_{j}\right)$                                         ▷ Change map cell state7:           **else**8:               $Restore\_unknown\_cell\_state(M_{i-1},c_{j})$9:               **if** $M_{i-1}\left(c_{j}\right)$ == unknown **then**10:                   $M_{i}\left(c_{j}\right)$ = unknown11:                   **for each** $c_{m}$ adjacent to $c_{j}$ **do**                               ▷ Check for objects borders cells state12:                       **if** $M_{i}\left(c_{m}\right)$ == free **then**13:                          $M_{i}\left(c_{m}\right)$ = occupied14:                       **end if**15:                   **end for**16:               **end if**17:           **end if**18:        **end for**19:    **end for**20:**end function**

### 5.6. Pose Updating

The *pose updating* module is activated when the *localisation check* detects that the robot is lost with no possibility to recover its pose (line 8 in Algorithm 1). To avoid possible collisions, when the localisation error is too high, the robot is stopped (line 2 in Algorithm 7) until the end of the pose-updating process (line 7 in Algorithm 7).

This module takes three inputs: the last estimated pose from the localization algorithm, denoted as $\tilde{X}R\left(k\right)$; the most recently updated map $M_{i}\left(k\right)$; and the current laser measurement Z(k). The objective is to determine the robot’s pose by comparing its current perception, represented by the laser measurements Z(k), with the expected point cloud Pexp(k) that would be observed if the robot were in the last correctly estimated pose ${\tilde{X}}_{R}\left(k\right)$. This expected point cloud is computed using the last updated map $M_{i}\left(k\right)$ based on the estimated pose (line 4 in Algorithm 7).

To achieve this, the *pose updating* module estimates the rigid transformation T(k) between the point cloud P(k) obtained from Z(k) (line 2 in Algorithm 7) and the point cloud $P_{exp}\left(k\right)$ computed from the map $M_{i}\left(k\right)$ using the last known correct pose $\tilde{X}R\left(k\right)$ (line 4 in Algorithm 7). The computation of Pexp(k) follows the same approach as described in Section 5.1. The estimation of T(k) can be achieved using various methods that find a rigid transformation between two point clouds, such as iterative closest point [29] or coherent point drift [30].
**Algorithm 7** Pose-updating function1:**function** Pose_Updating($Z\left(k\right),M_{i}\left(k\right),{\tilde{X}}_{R}\left(k\right)$)2:    Stop_robot_service()3:    P(k)=Compute_hit_P(K)(Z(K))4:    $P_{exp}=Compute\_P_{exp}\left(M_{i}\left(k\right),{\tilde{X}}_{R}\left(k\right)\right)$5:    $T\left(k\right)=Find\_Transform(P_{exp},P\left(k\right))$6:    Re_Initialise_Localisation_filter(T(k))7:    Start_robot_service()8:**end function**

## 6. Experiments and Simulations

Results of simulations and experimental data of the proposed approach are now reported. First, the procedure followed for both simulations and experiments is presented. Then, we provide an example of how the localisation check module works in case condition (6) occurs, and therefore, the map update is suspended. Subsequently, the results achieved in one hundred simulation scenarios are shown by giving a quantitative performance evaluation of the system in terms of map quality and localisation improvement. We compare the updated computed maps with their corresponding ground truth ones based on quantitative metrics. Moreover, localisation accuracy with and without our updated maps is analysed through the Evo Python Package [31] and the uncertain covariance matrix associated with the estimated pose. Next, we present an example of how the pose update module works, confirming the robustness of the method, and in the end, we report the validation results performed during experiments in a real-world environment.

The code developed for the experiments is available at https://github.com/CentroEPiaggio/safe_and_robust_lidar_map_updating.git (accessed on 12 May 2023), while the videos of the reported experiments are available in the Supplementary Materials.

### 6.1. Map Benchmarking Metrics

To evaluate the quality of our updated map with respect to the ground truth ones, we adopt, as in [7], three metrics, reported here for reader convenience. All the ground truth maps were built using the ROS Slam Toolbox package [32].

Let m(q) and $\tilde{m}\left(q\right)$ be the occupancy probability of the cells that contain the point *q* in the maps *M* and $\tilde{M}$, respectively. Furthermore, let ν be the number of cells in the maps, and

$$\begin{array}{cc} \langle M\rangle = & \;\frac{1}{\nu }\sum_{m(q)\in M}m\left(q\right),\\ \langle M\tilde{M}\rangle = & \;\frac{1}{\nu }\sum_{\begin{array}{c} m\left(q\right)\in M,\;\tilde{m}\left(q\right)\in \tilde{M} \end{array}}\left(m\left(q\right)\cdot \tilde{m}\left(q\right)\right),\\ \sigma (M)= & \;\sqrt{\frac{1}{\nu }\sum_{m(q)\in M}{\left(m\left(q\right)-\langle M\rangle \right)}^{2}}. \end{array}$$

(1) Cross-correlation (CC). The cross-correlation metric measures the similarity between two maps based on means of occupancy probabilities of the cells, and is given by

$$CC\left(M,\tilde{M}\right)=100\left(\frac{\langle M\tilde{M}\rangle -\langle M\rangle \cdot \langle \tilde{M}\rangle }{\sigma \left(M\right)\cdot \sigma \left(\tilde{M}\right)}\right),$$

(2) Map score (MS). The map score metric compares two maps on a cell-by-cell basis:

(10) $$MS\left(M,\tilde{M}\right)=100\left(1-\frac{1}{\nu }\sum_{\begin{array}{c} m(q)\in M\\ \tilde{m}\left(q\right)\in \tilde{M} \end{array}}{\left(m\left(q\right)-\tilde{m}\left(q\right)\right)}^{2}\right),$$

taking into account only cells that are occupied in at least one map to avoid favouring the map with large free space.

(3) Occupied picture-distance-function (OPDF). The occupied picture-distance-function metric compares the cells of a map *M* to the neighbourhood of the corresponding cells in the other map $\tilde{M}$, and vice versa. The occupied picture-distance-function can be computed as

(11) $$OPDF_{as}\left(M,\tilde{M}\right)=100\left(1-\frac{1}{\nu_{M}\cdot r}\sum_{i=1\ldots \nu_{M}}^{\nu_{M}}d_{i}\right),$$

where $\nu_{M}$ is the number of occupied map cells in *M*; $d_{i}$ is the minimum between the search space amplitude *r* (e.g., a rectangle of width *w* and height *h*, $r=\sqrt{w^{2}+h^{2}}$) and the Manhattan-distance of each occupied cell of the first map *M* to the closest occupied cell on the second map $\tilde{M}$. Since the function in (11) is not symmetric, we consider the average of the OPDF distance function from *M* to $\tilde{M}$ and from $\tilde{M}$ to *M*, as follows:

$$OPDF\left(M,\tilde{M}\right)=\frac{OPDF_{as}\left(M,\tilde{M}\right)+OPDF_{as}\left(\tilde{M},M\right)}{2}.$$

### 6.2. Simulation Design

All the experiments and examples conducted follow the same procedure:

1. The robot is immersed in an initial world, usually denoted with $W_{1}$, and it is teleoperated to build an initial static map $M_{1}$ through the ROS Slam Toolbox package. Given i=2, the proposed map update procedure starts from 2.
2. The world is changed to create $W_{i}$ similar to the previous world $W_{i-1}$.
3. The robot autonomously navigates in the new environment by localising itself with adaptive Monte Carlo localisation (AMCL) [33] using the previous static map $M_{i-1}$, while our approach provides a new updated map $M_{i}$.
4. We increase *i* by one and restart from 2.

For all the worlds created $W_{i}$ with $i=2,...,N_{W}$, a ground truth map $G_{i}$ is built for the map quality comparison.

### 6.3. Simulation Results

The laptop utilized for the simulations had an Intel Core i7-10750H CPU, 16 GB of RAM, and Ubuntu 18.04 as the operating system. To simulate a 290-square-meter industrial warehouse, we employed models from Amazon Web Services Robotics [34]. These models were employed within the Gazebo simulator [35]. The specific robot used in the simulation was the Robotnik XL-Steel platform, which was equipped with two SICK s300 Lidars (Robotnik xl-steel simulator, https://github.com/RobotnikAutomation/summit_xl_sim, accessed on 1 February 2021).

#### 6.3.1. Localisation Check

The goal of the first simulation is to show how the procedure described in Section 5.2, for which the map update system is suspended due to excessive localisation errors, works.

To fully understand the necessity of both map update interruption and localisation check introduction, we provide the comparison results with our old approach [7] in Appendix A.3.

**Figure 10:**
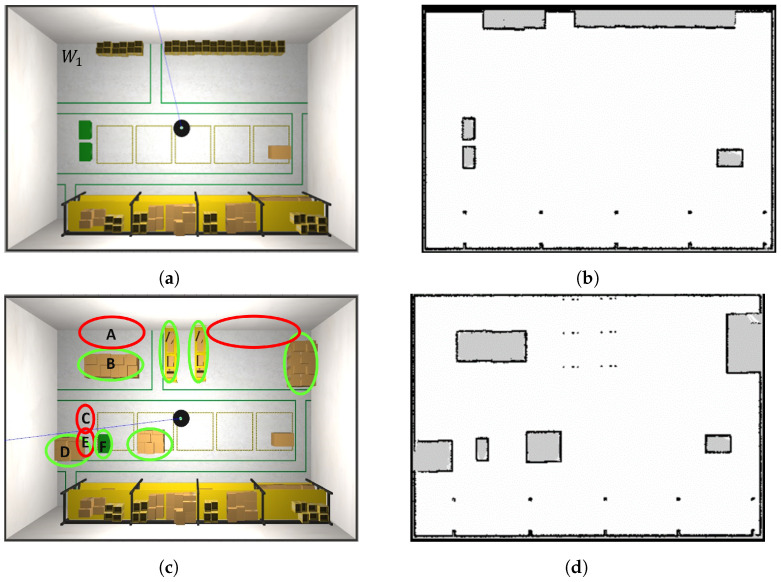
Scenario for the localisation check module testing. (**a**) Initial world $W_{1}$; (**b**) built map $M_{1}$, coincident with the ground truth $G_{1}=M_{1}$; (**c**) world $W_{2}$, whose map must be built based on $M_{1}$, the green and red circles are the objects added and removed respectively with respect to the world, while the letters A-F refer to the changes identified by the robot after around 10 s; (**d**) ground truth map $G_{2}$ of world $M_{2}$.

Let the robot operate in a world $W_{2}$, localising itself based on the map $M_{1}$ built while navigating in the previous world $W_{1}$, and let the proposed algorithm update the static map to provide $M_{2}$. Simulations are reported for the world $W_{1}$ reported in Figure 10a, with the corresponding built map $M_{1}$ in Figure 10b. In this case, we suppose that the map has been built correctly, and hence, it can be considered as a ground truth map, i.e., $G_{1}=M_{1}$. The changed worlds $W_{2}$ and its ground truth map $G_{2}$ are reported in Figure 10c,d, respectively, where green circles are the added obstacles and red circles are the removed ones.

**Figure 11:**
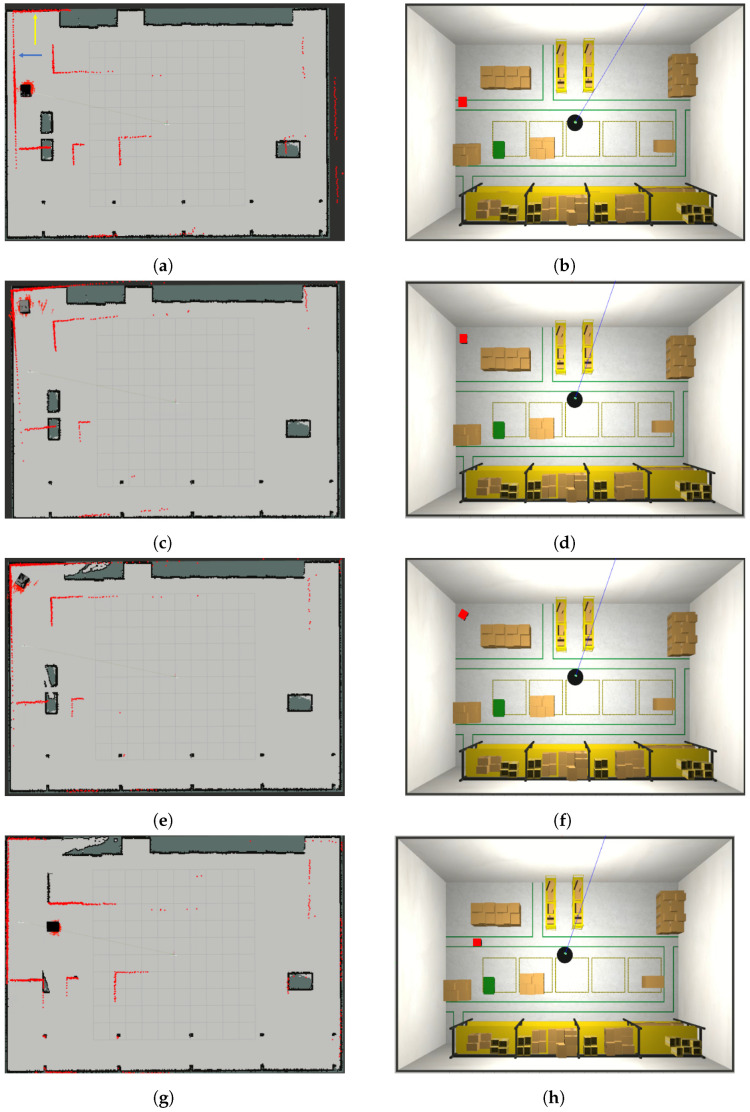
System evolution at the initial time (**a**,**b**), after 10 s (**c**,**d**), 20 s (**e**,**f**) and 60 s (**g**,**h**). (**a**) Updated map at time k=0, $M_{2}\left(0\right)$; (**b**) robot in $W_{2}$ at time k=0; (**c**) updated map $M_{2}\left(10\right)$; (**d**) robot in $W_{2}$ at time k=10; (**e**) updated map $M_{2}\left(20\right)$; (**f**) robot in $W_{2}$ at time k=20; (**g**) updated map $M_{2}\left(60\right)$; (**h**) robot in $W_{2}$ at time k=60.

The system evolution is reported in Figure 11, where the map update and the position of the robot in the world $W_{2}$ are reported at different time instants. In Figure 11a,b, the robot is immersed in the world $W_{2}$ with $X_{R}\left(0\right)=\left[2.0,9.5,0\right]$, while the localisation filter uses the map $M_{1}$ to localise the robot with an initial estimated pose ${\tilde{X}}_{R}\left(0\right)=\left[2.0,9.0,0\right]$. In Figure 11a, we can see our initial updated map, $M_{2}\left(0\right)=M_{1}$, and the robot in ${\tilde{X}}_{R}\left(0\right)$, with the particles of the localisation filter represented as red arrows close to the robot and the laser measurement Z(0) as red points. Observing Figure 11a, it is also possible to state that the robot is not localised correctly, because part of the laser measurements does not overlap with the edges of the map (see, e.g., the laser beams indicated by the blue arrow). However, the presence of a part of the measurements overlapping the map edges correctly (indicated by the yellow arrow) suggests that the localisation system can still recover the pose (case (6)). Under these conditions, the localisation check module suspends the map update, and the localisation error is kept monitored. The example in which the robot gets lost and therefore finds itself complying with condition (7) is shown in Appendix A.4.

Figure 11c,d represent the system after around 10 s: the localisation error is reduced as expected, and this is deduced by the fact that the laser beams at the top left of the map start to overlap the borders. However, the localisation error is still in the boundaries in (6), and hence, the map updating is still prevented. After 20 s, the situation is represented in Figure 11e,f, where the localisation system has recovered the right pose of the robot, and the map-updating process has been started. Indeed, the robot recognizes that boxes in ellipses A, C, and E (in Figure 10c) have been removed, and part of the corresponding cells have been set to free (light-gray in the figure). On the other hand, laser measurements (red points) detect the presence of boxes in ellipses B, D, and F (in Figure 10c). Finally, the algorithm proceeds for another 40 s, with the map-updating module working properly. The situation is represented in Figure 11g,h, where the previously detected left border of box B has been added to the map (cells in black).

#### 6.3.2. System Evaluation

**Figure 12:**
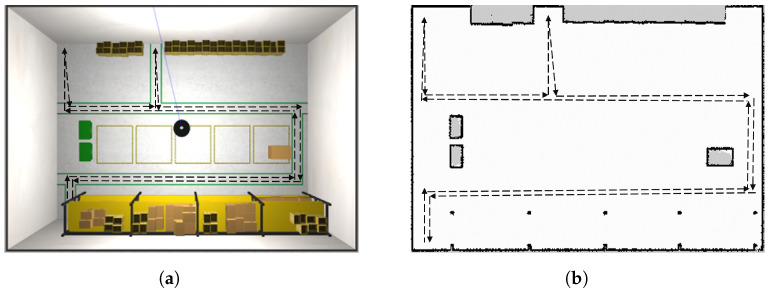
Autonomous trajectory path (black) in first world $W_{1}$ (**a**) and the relative initial map $M_{1}$ (**b**).

**Figure 13:**
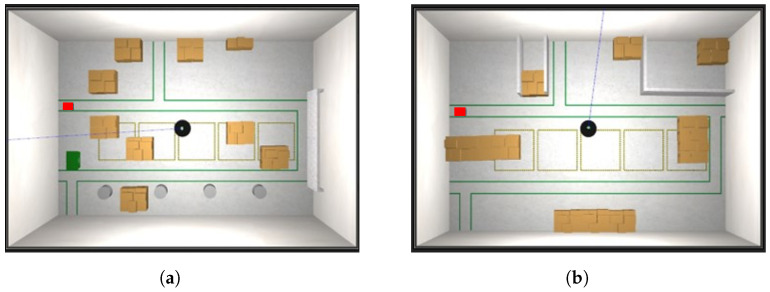
Warehouse gazebo environments for worlds 50 and 100. (**a**) World $W_{50}$; (**b**) world $W_{100}$.

To test the robustness of our algorithm for updating maps, we created one hundred variations of the same environment. The increasing changes were introduced to mimic how the arrangement of goods in a warehouse can evolve over time. In the initial environment, denoted as $W_{1}$, Figure 10a, the robot was manually operated to construct an appropriate initial map $M_{1}$. In the remaining environments, denoted as $W_{i}$, where *i* ranges from 2 to 100, the robot autonomously followed a predetermined trajectory (shown in Figure 12) to simulate the placement of materials within the warehouse. The 50th and final environments are depicted in Figure 13a,b, respectively.

Since we simulated a material deployment task in an industrial scenario, it is assumed that the initial pose of the robot is known, albeit with minor localisation errors. Thus, it is assumed that in all scenarios, the localisation system can recover the pose, and only condition (6) may occur.

To assess the effectiveness of the method, we obtained ground truth maps $G_{i}$ for each world $W_{i}$ (where *i* ranges from 1 to 100) using the Slam Toolbox.

(1) Updating Performance.

**Figure 14:**
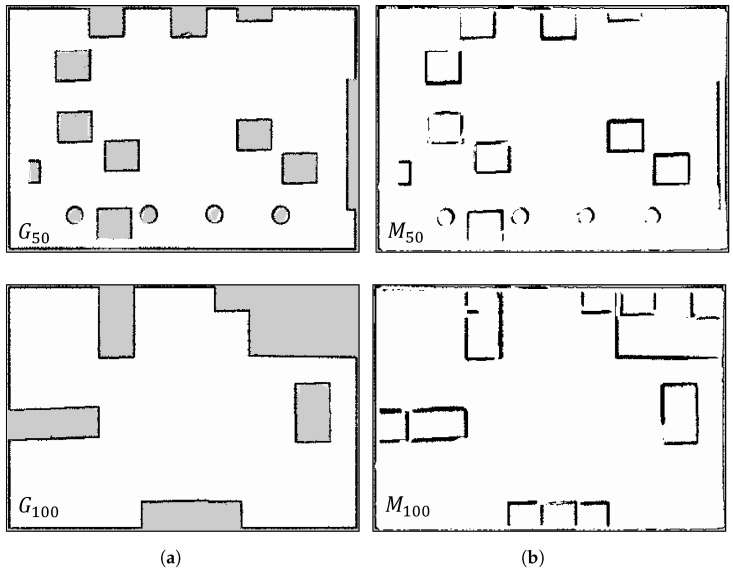
Map comparisons. (**a**) Ground truth; (**b**) proposed approach.

The initial map $M_{1}$ associated with $W_{1}$ and the planned trajectory can be seen in Figure 12 (all maps have dimensions of 13.95 m × 20.9 m with a resolution of 5 cm). A qualitative evaluation of our updating method is presented in Figure 14, which compares the ground truth maps $G_{i}$ (for *i* equal to 50 and 100) with their corresponding updated maps $M_{i}$ (also for *i* equal to 50 and 100). It is important to note that in the ground truth maps (depicted on the left side), the cells within obstacles are marked as unknown (light grey). However, in the maps $M_{50}$ and $M_{100}$, those cells are designated as free (white). This distinction arises because the obstacles were not present in the initial map $M_{1}$, but were later introduced. As a result, these cells are physically unobservable by the robot’s Lidar, and there is no need to update them since they do not impact autonomous navigation.

This qualitative comparison validates that our technique effectively detects environmental changes, with each map accurately reflecting the simulated scenario.

**Figure 15:**
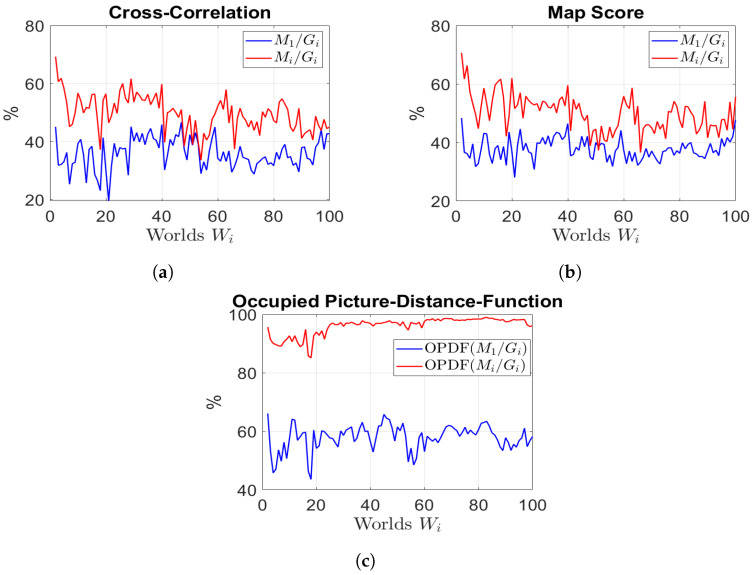
Quantitative map evaluation in each simulation scenario. (**a**) Cross-correlation metric results $M_{1}$/$G_{i}$ and $M_{i}$/$G_{i}$; (**b**) map score metric results $M_{1}$/$G_{i}$ and $M_{i}$/$G_{i}$; (**c** occupied picture-distance-function metric results $M_{1}$/$G_{i}$ and $M_{i}$/$G_{i}$.

To perform a quantitative assessment, we refer to Figure 15, which provides a comparison between the initial map $M_{1}$ and our updated maps $M_{i}$ (where *i* ranges from 2 to 100) in relation to the ground truth maps $G_{i}$ (where *i* ranges from 2 to 100). This evaluation employs the metrics described in Section 6.1, where a perfect correspondence between the two maps corresponds to a score of 100%.

In order to measure the differences between the current and initial environments, a map comparison is conducted between $M_{1}$ and $G_{i}$, as indicated by the blue data in Figure 15a–c. As expected based on each metric, the updated map $M_{i}$ consistently outperforms the initial map $M_{1}$ when compared to the ground truth. Indeed, the results achieved by employing the initial two metrics, CC and MS, indicate a level of similarity between the updated maps and the ground truths that surpasses the original map by approximately 20%. On the other hand, when considering the third metric, 0PDF, the improvement becomes even more pronounced, with a remarkable 40% increase in similarity. It is worth noting that not updating the obstacles’ internal cells affects the first two metrics, but not the third, which reports more accurate results.

Based on both qualitative and quantitative evaluations we are able to assess that our technique is able to effectively detect and represent environmental changes. Indeed, the updated maps accurately reflect the evolving scenario, providing valuable insights for autonomous navigation. It is worth noting that the predefined trajectory of the robot is not specifically designed to explore the warehouse area, but rather, to simulate item deployment. Consequently, there is a possibility that the measurements may overlook environmental changes that are not observable along the path of the robot’s movement.

(2) Localisation Performance.

To assess the enhancements in localisation performance resulting from the utilisation of updated maps, we compared the AMCL pose estimate based on two different maps: the initial map $M_{1}$ and the most recent available updated map $M_{i-1}$. These estimates were compared with the reference ground truth obtained from the simulator.

**Figure 16:**
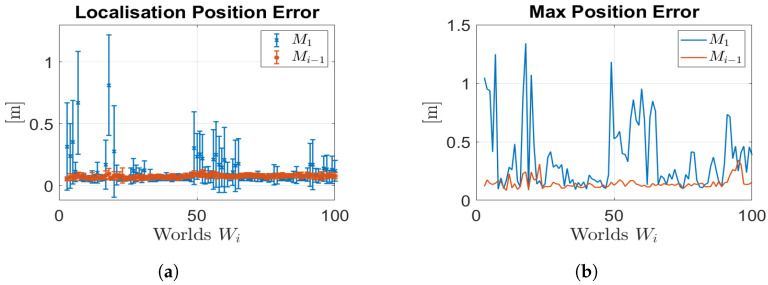
Localisation performance: localisation with old map $M_{1}$ (blue); localisation with last updated map $M_{i-1}$ (red). (**a**) Position errors; (**b**) max position errors.

**Figure 17:**
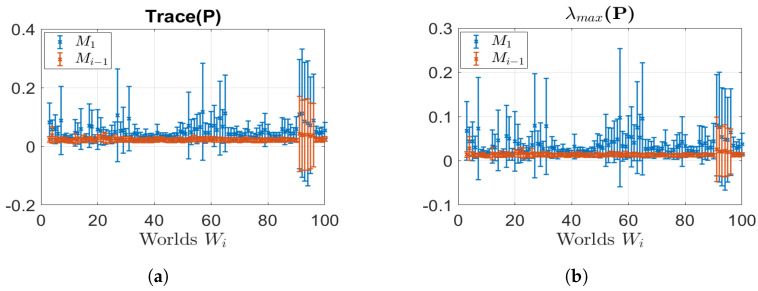
Estimated pose covariance matrix (P) results: localisation with old map $M_{1}$ (blue); localisation with last updated map $M_{i-1}$ (red). (**a**) Trace (P) comparison (μ,σ); (**b**) $\lambda_{max}\left(P\right)$ comparison (μ,σ).

The results are shown in terms of mean (μ) and variance (σ) for each world. Figure 16 depicts the comparison of both the estimated position errors and the maximum position errors. As anticipated, utilising an updated map led to a reduction in localisation errors. Indeed, the use of the updated map yields errors that consistently stay below 10 cm, with minimal variance and a maximum position error of 40 cm. Conversely, relying on the initial map leads to localization errors that can exceed 50 cm, exhibiting a high level of variance and a maximum error of over one meter. Furthermore, we examined the uncertainty associated with the estimated robot pose by calculating the trace and the maximum eigenvalue (λ) of the covariance matrix (P) related to the estimated pose. As illustrated in Figure 17, the utilisation of an updated map significantly decreased the uncertainty in the robot’s pose, since the trace decreased from approximately 0.05 to around 0.03, while the maximum eigenvalue decreased from 0.03 to 0.015. Importantly, we emphasize that despite the presence of localisation errors, the localisation check played a critical role in achieving excellent results in terms of map updating. This indicates that our technique successfully combines localisation and map updating, contributing to more accurate and reliable representations of the environment. These analytical results provide valuable insights into the effectiveness of utilising updated maps for localisation and highlight the importance of the localisation check in achieving superior map-updating outcomes.

(3) Hardware Resource Consumption.

**Figure 18:**
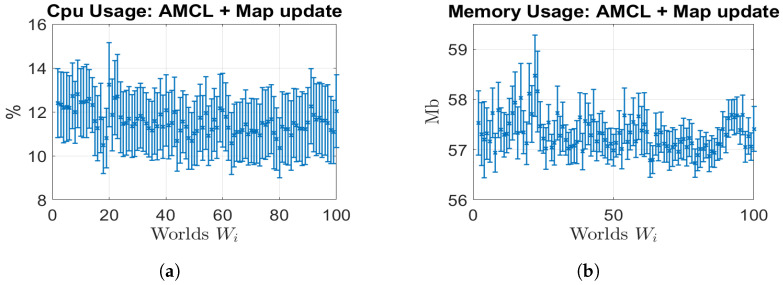
Resource consumption. (**a**) CPU usage; (**b**) memory usage.

In this section, we provide the hardware resources of the CPU percentage and memory MB utilisation computed by the ROS package “cpu_monitor” (cpu_monitor, https://github.com/alspitz/cpu_monitor, accessed on 20 September 2021) during the map-updating and localisation phases in terms of μ and σ. These metrics provide insights into the computational demands of our proposed solution. Figure 18a depicts the percentage of CPU used in each simulation, whereas Figure 18b depicts the memory utilisation per map update. The results highlight that our proposed solution operates as a memory-limited algorithm, which makes it well-suited for long-term operations. The CPU usage remains within acceptable limits, 10–13%, ensuring efficient utilization of computational resources. Moreover, the memory utilization of around 57.5 Mb demonstrates a stable pattern throughout the map-updating process, indicating that our solution effectively manages memory allocation.

The significance of these analytical results lies in understanding the resource demands of our algorithm. By demonstrating that our solution is memory-limited and operates within reasonable CPU percentages, we provide evidence of its practical feasibility and suitability for prolonged operations.

These insights into the hardware resource utilization further support the robustness and efficiency of our proposed solution, contributing to its overall significance in real-world scenarios.

#### 6.3.3. Pose Updating

In this section, two cases are reported to show how the pose-updating algorithm works and makes the map-updating process more robust. In both cases, during robot navigation, to activate the pose-updating module, the localisation error is forced in the scenario by manually setting a wrong robot’s estimated pose through a dedicated ROS service.

**Figure 19:**
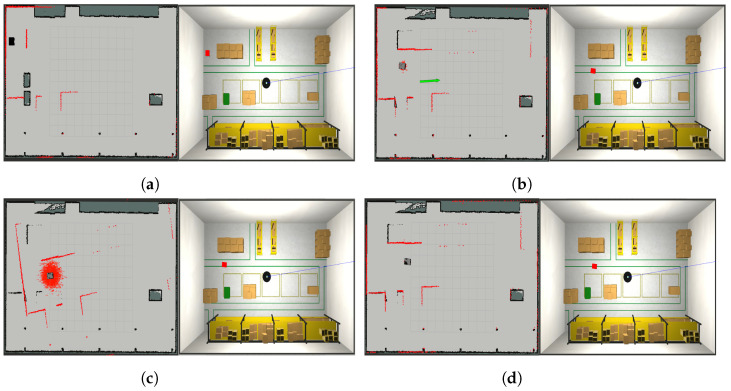
Second-case scenario: the map used by the robot to locate itself does not reflect the current environment $M_{1}\neq G_{2}$. (**a**) Initial configuration; (**b**) the robot’s estimated pose is manually reinitialized, green arrow; (**c**) the localisation error is too big, and the pose-updating module is activated; (**d**) the pose-updating module recovers the robot’s estimated pose.

In the first case, the algorithm is tested in a condition where the map used by the robot to localise itself reflects the current environment $W_{2}$, i.e., $M_{2}\equiv G_{2}$. This experiment was conducted to assess the performance of the pose-updating algorithm module in nominal conditions, e.g., the best-case scenario, and the qualitative results can be found in Appendix A.5. In the second case, instead, we tested the algorithm using a possibly incorrect map as the proposed approach described in Section 6.2. Indeed, the robot uses the previously updated map $M_{1}$ to localise itself, while the localisation check and the map-updating and pose-updating modules are active to produce an updated map $M_{2}$. The whole evolution of the system is reported in Figure 19, where the last built map $M_{1}$ does not reflect the current world $W_{2}$, i.e., $M_{1}\neq G_{2}$, as visible in Figure 19a. After a while, the robot’s estimated pose is manually changed through the ROS service (see the green arrow in Figure 19b), and the map-updating process is suspended due to the high localisation errors introduced. Once the robot’s estimated pose becomes even larger (see red arrows in Figure 19c), the *pose update* module is activated, and it recovers the right global robot pose, Figure 19d, and the map-updating process can be restarted. As can be appreciated from Figure 19c,d the maps are the same, and hence have not been corrupted by the presence of localisation errors.

### 6.4. System Validation

**Figure 20:**
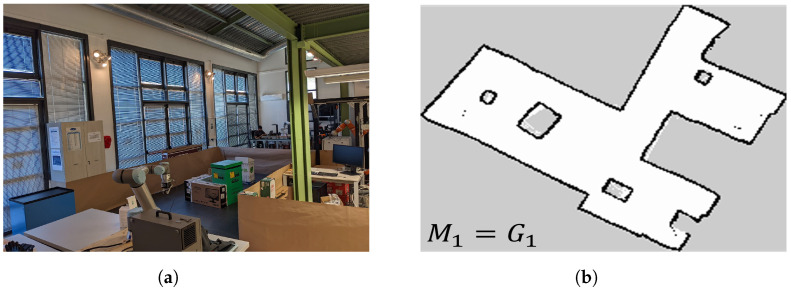
Lab environment for the experiments. (**a**) Initial environment; (**b**) map $M_{1}$.

To validate the newly proposed system in a real-world setting, we utilised bag files containing sensor measurements and odometry topics from our previous work. The experiments were conducted in a laboratory environment (depicted in Figure 20a) using a Summit-XL-Steel mobile platform equipped with two 2D-Lidar Hokuyo-UST-20LX sensors.

Four distinct environments were recreated by introducing changes in the positions of obstacles. The testing environment had an approximate size of 80 square meters. The robot constructed the initial map $M_{1}$ (shown in Figure 20b), and the ground truth maps $G_{i}$ (where *i* ranges from 2 to 4) were obtained through Slam Toolbox while performing teleoperated navigation at a speed of 0.15 m/s.

The performance of map updates and the utilization of hardware resources are quantified as in simulation. Since there was no external ground truth tracking system available, we compared the uncertainty in the estimated pose obtained using both the initial map $M_{1}$ and the most recently updated maps $M_{i-1}$ to evaluate the localisation performance. Given the real-world conditions, the localisation system was initialized with a robot pose in close proximity to the true pose.

#### 6.4.1. Updating Performance

**Figure 21:**
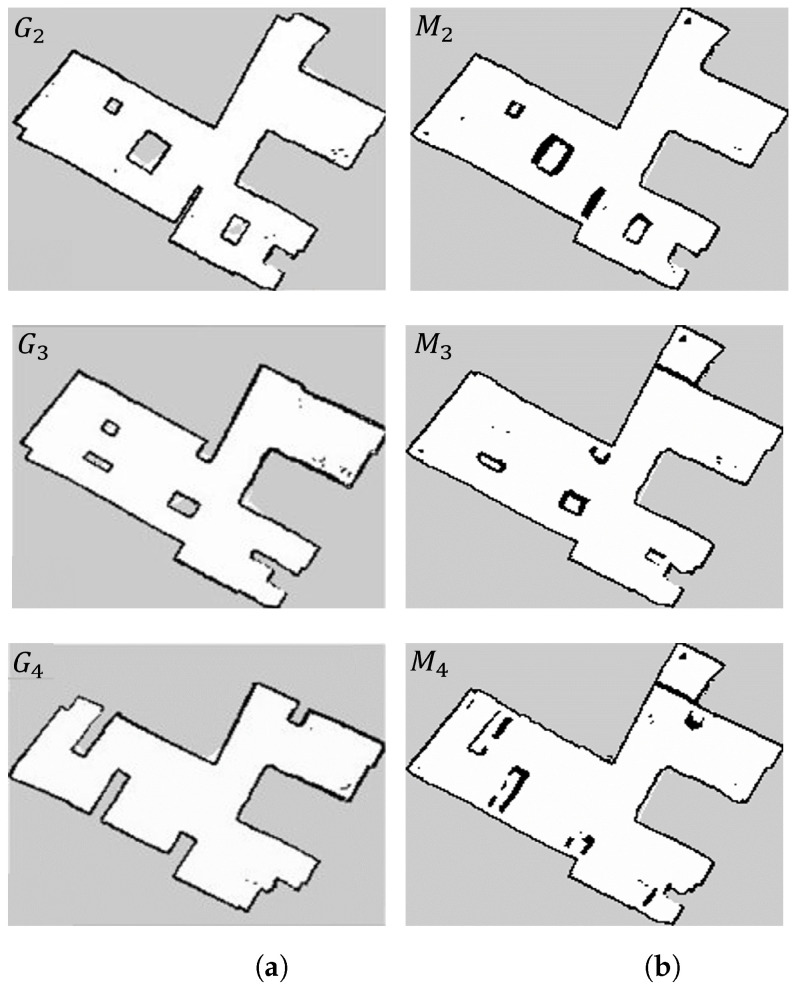
Map comparisons. (**a**) Ground truth; (**b**) proposed approach.

The qualitative and quantitative outcomes can be observed in Figure 21 (all maps have dimensions of 10.5 m × 8.55 m with a resolution of 5 cm) and Table 3. The observations made in Section 6.3.2 regarding the metric findings are applicable in this context as well. Despite the presence of noisy real data and higher localisation errors, the proposed method consistently generates updated maps that surpass the initial map when compared to ground truths. This highlights the capability of the system to dynamically add and remove obstacles without affecting the walls, thanks to the localisation check.

#### 6.4.2. Localisation Performances

**Figure 22:**
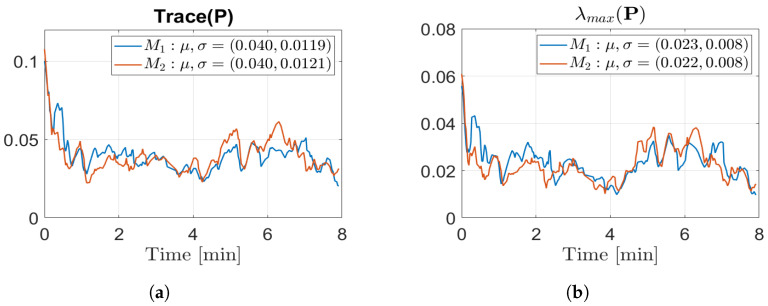
Estimated pose covariance matrix (P) results in $W_{3}$: localisation with old map $M_{1}$ (blue); localisation with last updated map $M_{2}$ (red). (**a**) Trace (P) comparison; (**b**) maximum eigenvalue comparison.

The analysis of localisation uncertainty was conducted in worlds $W_{3}$ and $W_{4}$. Since we obtained similar results, in Figure 22, only the analysis related to $W_{3}$ is reported. The one referring to $W_{4}$ can be found in Appendix A.6 for completeness. Regarding Figure 22, we must stress that the low improvement percentage is due to the similarity between the worlds. Unfortunately, it was not possible to increase the differences between worlds, due to the robot dimensions with respect to the available area. However, the results are encouraging, and validate the approach, even in dynamic real-world environments.

#### 6.4.3. Hardware Resource Consumption

**Figure 23:**
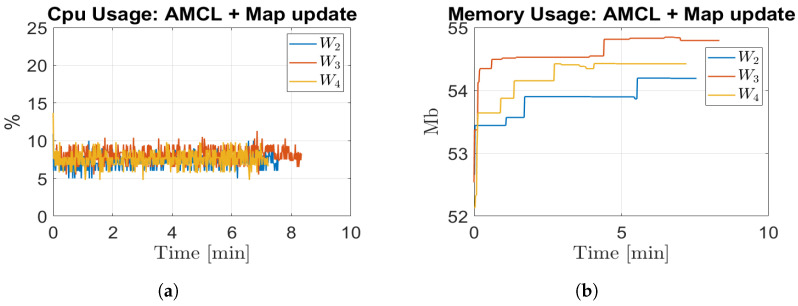
Resource consumption. (**a**) CPU usage; (**b**) memory Usage.

The CPU utilisation and memory usage are illustrated in Figure 23. Due to the smaller size of the real environment and the limited number of environmental changes compared to the simulation, the CPU consumption decreases from 15% to 7%, and the memory usage decreases from 55–58 Mb to 52–55 Mb. These reductions are observed when comparing the real environment to the simulated environments.

## 7. Discussion

The aim of this work was to extend our previous work in order to update the map robustly with respect to big localisation and measurement errors. Although our previous method gave promising results in terms of map quality and localisation improvement, the big limitation of that approach is that without a perfect localisation system, the map-updating process provides a corrupted map (see Appendix A.3). We have, therefore, developed a safety mechanism to pause the map update in the case of big localisation errors. This mechanism is related to the number of detected changes measurements in the current environment compared to the previous map, as shown in Section 6.3.1. As numerical validation confirmed, the mechanism was sufficient to prove both the map quality and localisation improvement. It is worth noting that to the authors’ best knowledge, as in all other available approaches, in case of substantial changes in the environment, the proposed system would detect numerous detected change measurements and would not update the map, even in the absence of localisation errors. The only solution, in this case, would be to recompute the map from scratch.

From the hardware resource consumption point of view, the proposed algorithm does not require high computation capabilities. Indeed, differently from other approaches such as those based on graphs, the resource consumption does not depend on the robot’s travelled path but only on the laser scan measurements processing. In this case, the CPU usage can be further significantly reduced by simply discarding some range measurements from the processed laser scan. Moreover, the memory usage depends only on the number of changed cells and the size $N_{b}$ of the buffers, and not on the environment dimension. Concluding, as both the numerical validation and real-data experiments confirmed, the proposed memory-limited solution is suitable for lifelong operation scenarios.

So far, we used only the Lidar measurements to update the map, since they are the most common sensors used to build 2D occupancy grid maps in industrial applications such as logistics and surveillance, where the problem of having an updated map for autonomous navigation is still an issue. In future work, we plan to extend our system to also manage 3D sensors in order to update the 3D occupancy map for all autonomous mobile robots that navigate based on occupancy grid maps. The extension to 3D map-updating algorithms will allow for the use of such approaches beyond logistics/industrial applications and ground robots, e.g., to legged robots and UAVs. Moreover, the validation of the localisation performance in a real environment could be improved with an external tracking system as a benchmark.

## 8. Conclusions

In the present study, we proposed a robust strategy for dealing with dynamic situations in long-term operations based on occupancy grid maps. The goal was to improve our prior work in order to update the map reliably in the face of large localisation and measurement mistakes. Extensive simulations and real-world trials have been used to validate the approach. Because of the fail-safe technique devised, the updated maps exhibit no symptoms of drift or inconsistency, even when the localisation error is relatively substantial. Furthermore, they mirror the setup of the environment and improve the AMCL localisation performance in simulations. In addition, we proved that our system is able to correctly detect when map updating should be suspended, and, if the robot is lost, update the estimated robot pose accordingly. Simulations and experiments were conducted to validate the approach in different and challenging dynamic environments.

## Figures and Tables

**Table 1:** List and description of the variables of interest used in the paper.

Variable Name	Description
R	Robot
$X_{R}\left(k\right)$	Robot pose
p(k)	Robot position at time *k*
θ(k)	Robot orientation at time *k*
${\tilde{X}}_{R}\left(k\right)$	Robot estimated pose
$\tilde{p}\left(k\right)$	Robot estimated position at time *k*
$\tilde{\theta }\left(k\right)$	Robot estimated orientation at time *k*
$\epsilon_{d}$	Robot pose displacement threshold
$W_{i}$	*i*-th Robot World
$M_{i}$	Occupancy grid map of $W_{i}$
$c_{j}\left(q\right)$	*j*-th map’s cell associated to a Cartesian position *q*
$\left(x_{map},y_{map}\right)$	Origin of the grid coordinates
(u,v)	Grid coordinates of a point p=(x,y)
Z(k)	Laser range measurement at time *k*
$z_{i}\left(k\right)$	*i*-th laser beam in Z(k)
*n*	Number of laser beams in a laser measurement
P(k)	Point cloud associated to Z(k)
$p_{i}\left(k\right)$	*i*-th hit point at time *k* belongs to P(k).
$x_{i},\;y_{i}$	Cartesian coordinates of $p_{i}$
$\theta_{0}$	Angular offset of the first laser beam with respect to the orientation of the laser scanner
Δθ	Angular rate between adjacent beams
$C_{p_{i}}=\left\{\begin{array}{c} c_{1},\ldots ,c_{\mu } \end{array}\right\}$	The set of cells passed through by the measurement associated to $p_{i}$
$c_{\mu }=c\left(p_{i}\right)$	The cell associated to $p_{i}$.
$p_{c_{j}}$	point in the centre of the cell $c_{j}$
$B_{c_{j}}$	Rolling buffer of $c_{j}$
$N_{b}$	Size of $B_{c_{j}}$
$z_{e,i}\left(k\right)\in Z_{e}$	Expected value for the *i*-th laser beam $z_{i}\left(k\right)\in Z\left(k\right)$
$p_{e,i}\left(k\right)$	Expected hit point associated with $z_{e,i}\left(k\right)$
$\left(x_{e,i},y_{e,i}\right)$	Cartesian coordinates of $p_{e,i}\left(k\right)$
$P_{e,i}\left(k\right)$	Set of expected hit points
$v<\frac{\Delta \theta }{n_{e}}$	Perturbation
$D_{t}h$	Distance threshold, function of $z_{i}$
$n_{dc}$	Number of the “detected change” measurements
$n_{min}$	Min threshold related to localisation error to suspend the map-updating process, function of *n*
$n_{max}$	Max threshold related to localisation error to update robot pose, function of *n*
$p_{t}h$	Threshold point belonging to a laser beam. $p_{t}h=m\tilde{p}+\left(1-m\right)p_{i}$
tol	Distance threshold, function of $z_{i}$
$n_{c_{j}}$	Counter of “changed” flag in $B_{c_{j}}$
$P_{exp}\left(k\right)$	Expected point cloud computed from the last updated map $M_{i}\left(k\right)$
T(k)	Rigid transformation between P(k) and $P_{exp}\left(k\right)$
$G_{i}$	*i*-th ground truth map

**Table 2:** List, descriptions, and values of the parameters of interest used in the paper.

Parameter Name	Description	Value
$N_{b}$	Size of $B_{c_{j}}$	10
$n_{e}\in N_{0}$	Design parameter	1
$N=2n_{e}+1$	Number of points in $P_{e,i}\left(k\right)$	3
*l*	Design parameter $l=-n_{e},\;\ldots ,\;0,\;\ldots ,\;n_{e}$	[−1, 0, 1]
$D_{\epsilon_{min}}$	Minimum fraction of acceptable changed measurements with respect to the number of laser beams that allows a good localisation performance	0.75
$D_{\epsilon_{max}}$	Maximum fraction of acceptable changed measurements with respect to the number of laser beams, in addition to which the robot has definitively lost its localisation.	0.90
$\epsilon_{n_{c_{j}}}$	Threshold for $n_{c_{j}}$	7
$\epsilon_{p}$	Robot linear displacement threshold	0.05 [m]
$\epsilon_{\theta }$	Robot angular displacement threshold	20${}^{\circ }$

**Table 3:** Quantitative map evaluation.

	$W_{2}$		$W_{3}$		$W_{4}$	
	$M_{1}/G_{2}$	$M_{2}/G_{2}$	$M_{1}/G_{3}$	$M_{3}/G_{3}$	$M_{1}/G_{4}$	$M_{4}/G_{4}$
CC (%)	69.69	78.95	63.77	68.80	60.87	67.14
MS (%)	54.63	74.57	51.44	72.58	50.45	70.26
OPDF (%)	84.61	97.25	78.92	95.37	82.18	94.33

**Figure A1:**
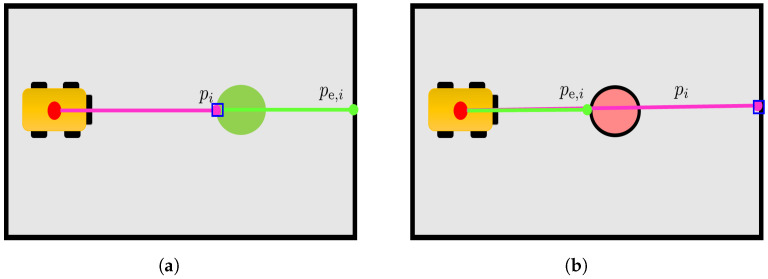
Ideal case where no localisation error occurs: by checking the current state of the cell $c(p_{i})$ associated to the hit point $p_{i}$, we may distinguish between (**a**) added obstacle if $state(c\left(p_{i}\right))=\mathrm{free}$ in $M_{i-1}$; (**b**) removed obstacle if $state(c\left(p_{i}\right))=\mathrm{occupied}$ in $M_{i-1}$. (**a**) Ideal case of added obstacle; (**b**) ideal case of removed obstacle.

**Figure A2:**
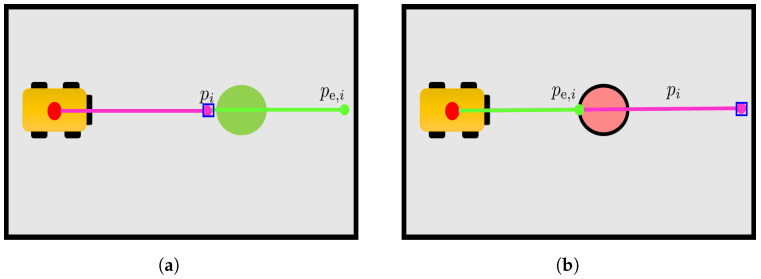
Case in which localisation error may occur: by checking the current state of the cell $c(p_{i})$ associated to the hit point $p_{i}$, we may not distinguish between (**a**) added obstacle with $state(c\left(p_{i}\right))=\mathrm{free}$ in $M_{i-1}$ and (**b**) removed obstacle with $state(c\left(p_{i}\right))=\mathrm{free}$ in $M_{i-1}$. (**a**) Case of added obstacle with localisation errors; (**b**) case of removed obstacle with localisation errors.

**Figure A3:**
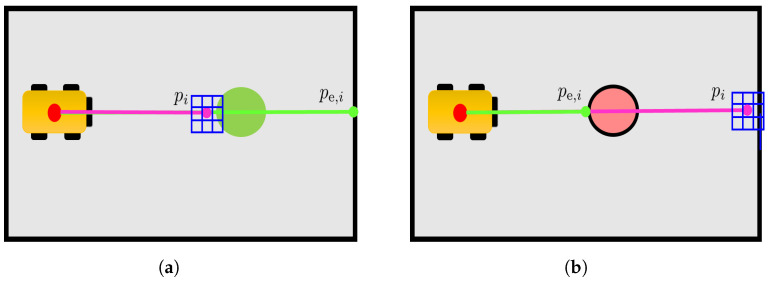
Considering cells sufficiently close to $c(p_{i})$, the system is able to distinguish between an added obstacle case from a removed obstacle one. Case (**a**): an obstacle has been added, and all cells close to $c(p_{i})$ are *free* in $M_{i-1}$. Case (**b**): an obstacle has been removed, and some cells close to $c(p_{i})$ are *occupied* in $M_{i-1}$. (**a**) Check of multiple cells in case of added obstacle; (**b**) check of multiple cells in case of removed obstacle.

**Figure A4:**
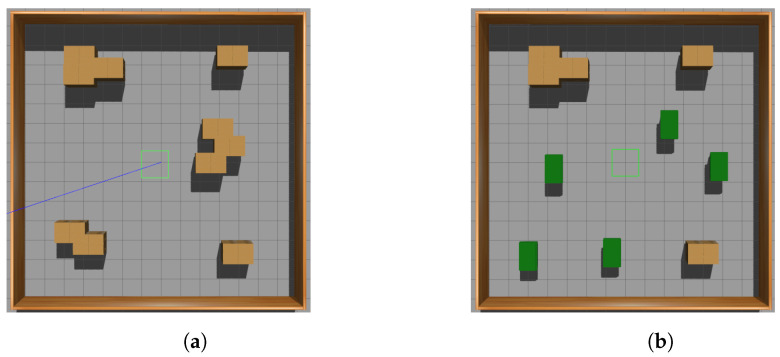
Scenario for comparison of active localisation check module vs. an always active map-updating process. (**a**) Initial $W_{1}$; (**b**) world $W_{2}$, whose map must be built based on $M_{1}$.

**Figure A5:**
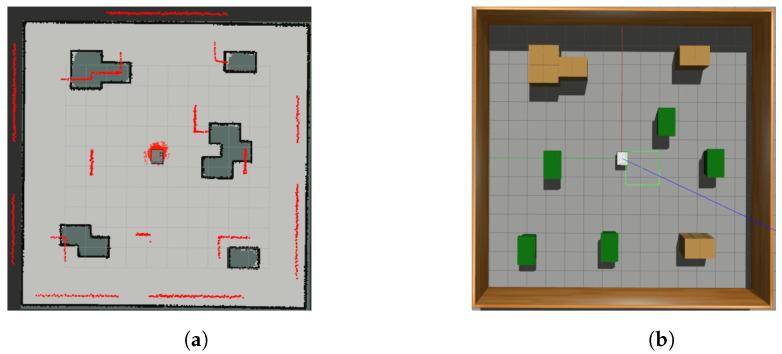
Initial example configuration. (**a**) Updated map $M_{2}\left(0\right)$, at time k=0, $M_{2}=M_{1}$; (**b**) robot in $W_{2}$ at time k=0.

**Figure A6:**
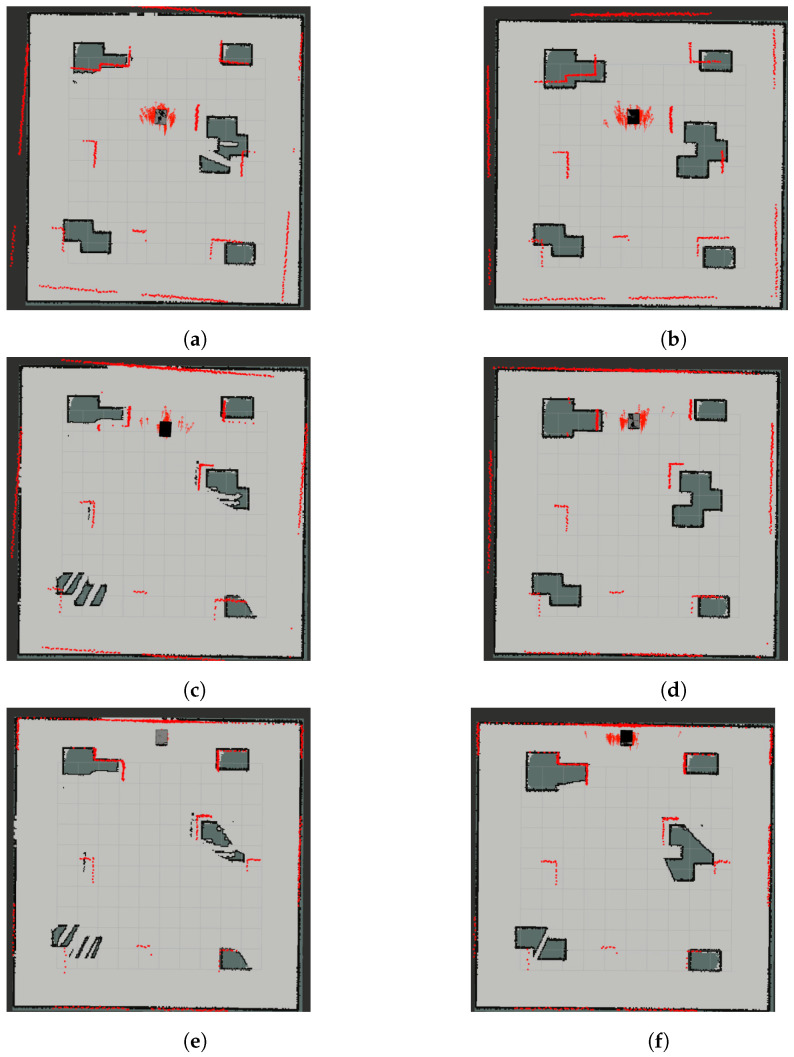
Updated mapping approaches comparison. (**a**) $M_{2}$ at time t=10s with active map-updating process; (**b**) $M_{2}$ at time t=10s with localisation check that prevents the map-updating process due to localisation errors; (**c**) $M_{2}$ at time t=20s with active map-updating process; (**d**) $M_{2}$ at time t=20s with localisation check that prevents the map-updating process due to localisation errors; (**e**) $M_{2}$ at time t=30s with active map-updating process; (**f**) $M_{2}$ at time t=30s with localisation check that activates the map-updating process.

**Figure A7:**
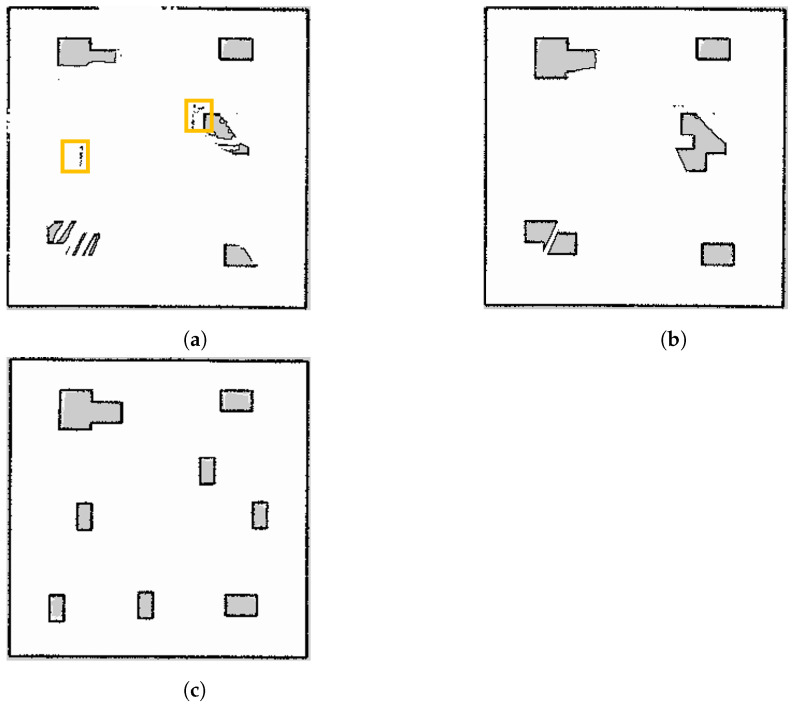
Maps obtained with the two approaches and the corresponding ground truth $G_{2}$. (**a**) Map $M_{2}$ built with an always-active map-updating process; (**b**) map $M_{2}$ built with the proposed approach that may prevent the map-updating process in the case of localisation errors; (**c**) ground truth map $G_{2}$ of world $M_{2}$.

**Figure A8:**
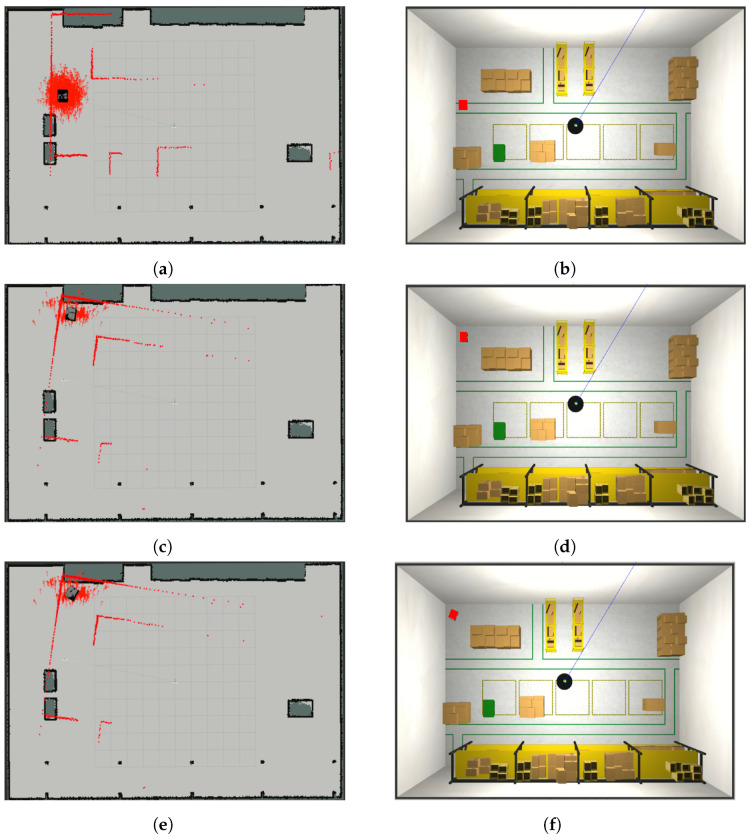
System evolution at the initial time (**a**,**b**) and after 10 s (**c**,**d**) and 20 s (**e**,**f**). (**a**) Updated map $M_{2}\left(0\right)$; (**b**) $W_{2}\left(0\right)$; (**c**) the map $M_{2}\left(10\right)$ not changed with respect to $M_{2}\left(0\right)$ do to high localisation errors; (**d**) $W_{2}\left(10\right)$; (**e**) updated map $M_{2}\left(20\right)$; (**f**) $W_{2}\left(20\right)$.

**Figure A9:**
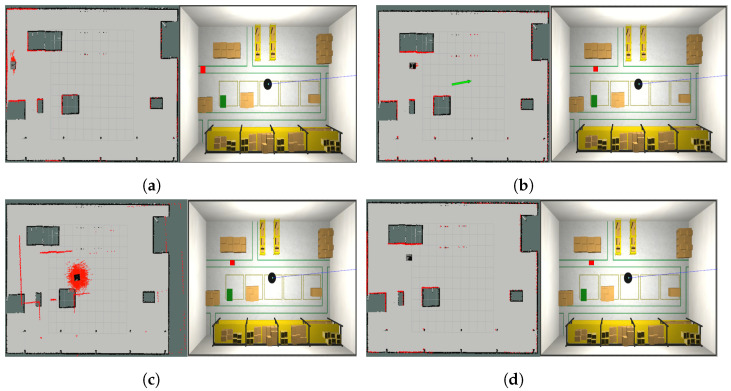
First-case scenario: the map used by the robot to locate itself reflects the current environment, i.e., $M_{2}\equiv G_{2}$. (**a**) Initial configuration; (**b**) the robot’s estimated pose is manually reinitialized (green arrow); (**c**) the localisation error is too big, and the pose-updating module is activated; (**d**) the pose-updating module recovers the robot’s estimated pose.

**Figure A10:**
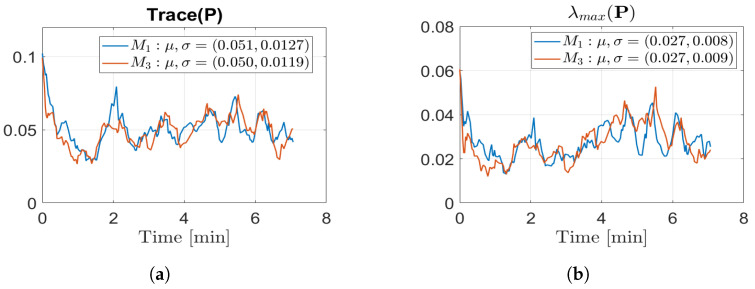
Estimated pose covariance matrix (P) results in $W_{4}$: localisation with old map $M_{1}$ (blue); localisation with last updated map $M_{3}$ (red). (**a**) Trace (P) comparison; (**b**) maximum eigenvalue comparison.

## Data Availability

The code used to realize the reported experiments can be found at https://github.com/CentroEPiaggio/safe_and_robust_lidar_map_updating.git (accessed on 12 May 2023), while the videos of the reported experiments are available in the Supplementary Materials.

## Supplementary Materials

The following supporting information can be downloaded at: https://www.mdpi.com/article/10.3390/s23136066/s1, Supplementary Video: Experiment for Safe and Robust Map Updating for Long-Term Operations in Dynamic Environments. It shows the execution and the obtained results of the experiments reported in the manuscript.

## Appendix A

### Appendix A.1

To correctly classify the “detected change measurement”, we choose a linear function for the distant threshold that considers the localisation and measurement noise errors. Indeed, due to these errors, there will always be a certain distance between a measured hit point $p_{i}$ and the closest expected hit point $p_{e,i}$. An incorrect choice of $D_{t}h$ can lead to a wrong classification, and the error in the estimated yaw $\tilde{\theta }$ can affect this classification process. From (1), it is clear that an error δθ∈R in the yaw estimation will produce a certain error $\delta p_{i}\in R^{2}$ in the computation of the measured hit point $p_{i}$ in a fixed frame. The true yaw $\bar{\theta }\in R$ and the true hit point ${\bar{p}}_{i}\in R^{2}$ are defined as

(A1) $$\begin{array}{cc} \; & \bar{\theta }=\tilde{\theta }+\delta \theta , \end{array}$$

(A2) $$\begin{array}{cc} \; & {\bar{p}}_{i}=p_{i}+\delta p_{i}. \end{array}$$

From (1), (A1), and (A3):

(A3) $$\begin{array}{cc} \delta p_{i} & ={\bar{p}}_{i}-p_{i}=\\ \; & =\tilde{p}+z_{i}\left(\begin{array}{c} \cos(\theta_{0}+i\;\Delta \theta +\tilde{\theta }+\delta \theta )\\ \sin(\theta_{0}+i\;\Delta \theta +\tilde{\theta }+\delta \theta ) \end{array}\right)+\\ \; & -\tilde{p}-z_{i}\left(\begin{array}{c} \cos(\theta_{0}+i\;\Delta \theta +\tilde{\theta })\\ \sin(\theta_{0}+i\;\Delta \theta +\tilde{\theta }) \end{array}\right)=\\ \; & =z_{i}\left(\begin{array}{c} \cos\left(\theta_{0}+i\;\Delta \theta +\tilde{\theta }+\delta \theta \right)-\cos\left(\theta_{0}+i\;\Delta \theta +\tilde{\theta }\right)\\ \sin\left(\theta_{0}+i\;\Delta \theta +\tilde{\theta }+\delta \theta \right)-\sin\left(\theta_{0}+i\;\Delta \theta +\tilde{\theta }\right) \end{array}\right)=\\ \; & =z_{i}\left(\begin{array}{c} -2\sin\left(\frac{\delta \theta }{2}\right)\sin\left(\frac{\delta \theta }{2}+\theta_{0}+i\;\Delta \theta +\tilde{\theta }\right)\\ 2\sin\left(\frac{\delta \theta }{2}\right)\cos\left(\frac{\delta \theta }{2}+\theta_{0}+i\;\Delta \theta +\tilde{\theta }\right) \end{array}\right). \end{array}$$

Finally, assuming that the yaw estimation error δθ is small:

$$|\delta p_{i}|=z_{i}\cdot 2\left|\sin\frac{\delta \theta }{2}\right|\approx z_{i}\cdot \left|\delta \theta \right|.$$

Since the effect of an error on the yaw estimation is amplified by the measured range, the threshold $D_{t}h$ can be taken as a function of the measured range $z_{i}$. Hence, the choice of a saturated linear function:

$$D_{t}h\left(z_{i}\right)=\min\left(m_{t}h\cdot z_{i}+q_{t}h,\;D_{max}\right)$$

where $m_{t}h\in R^{+}$, $q_{t}h\in R^{+}$, $D_{m}ax\in R^{+}$. In this way, hit points associated with small range measurements are more likely to be flagged as ‘inconsistent with the map’ detecting tiny changes. On the other side, the classification of hit points associated with big range measurements is more robust to yaw estimation error.

### Appendix A.2. Change Detection of c(p i ) Integration

The state of $c(p_{i})$ must also be evaluated. However, in this case, the events of a newly added obstacle and of a removed one must be distinguished. Indeed, in an ideal situation, when a new obstacle is added, the hit point $p_{i}$ lies on the new obstacle, and hence, its associated cell $c(p_{i})$ has a *free* state in $M_{i-1}$, and hence, it should be changed to *occupied* (see Figure A1a). On the other hand, when an obstacle is removed, the hit point $p_{i}$ lies on another obstacle or part of the environment; hence, its associated cell $c(p_{i})$ has an *occupied* state in $M_{i-1}$, and hence, it should not be changed (see Figure A1b).

Unfortunately, in the case of localisation errors, it may occur that the (not ideally computed) cell $c(p_{i})$ corresponding to the measured hit point may be *free* in both cases of added or removed obstacles (see Figure A2). Hence, in the case of localisation errors, the distinction among added or removed obstacles is not possible with a direct evaluation of single-cell occupancy. On the other hand, in the case of an added obstacle, all cells sufficiently close to $c(p_{i})$ are also *free* in $M_{i-1}$, while in case of a removed one, some of those cells would be *occupied*, and hence, a distinction between the two cases is possible if the closed cells’ state is also checked (see Figure A3) .

To effectively detect an added obstacle, a new “changed” flag is added to the buffer of $c(p_{i})$ if the states of the neighbouring cells satisfy a certain condition. This condition is defined by Equation (9) in the text. It states that the state of cell c(q) should be “free” for all points *q* within a certain distance tolerance $tol(z_{i})$ from the hit point $p_{i}$. The tolerance $tol(z_{i})$ is a function of the measured range and is determined by the parameter *D*th in Equation (4).

### Appendix A.3. Continue Map Updating vs. Localisation Check On

To fully understand the necessity of such an interruption in the map-updating process, we provide the comparison results of our old approach [7] (where the map was always updated) with the one proposed in this paper with an initial localisation error. The comparison is performed in the scenario reported in Figure A4: the first environment $W_{1}$ is in Figure A4a, while the robot is operating in world $W_{2}$ (Figure A4b).

In this example, the robot is navigating in world $W_{2}$; the localisation algorithm will use $M_{1}$ to provide the global estimated robot pose, while both approaches will update the map providing $M_{2}$. As can be deduced from the red arrows and the not-overlapping measurements with map borders in Figure A5, we intentionally misinitialised the localisation filter with an error of around 0.7 m. The robot will move in a straight line until the localisation algorithm recovers the right pose.

In the left column of Figure A6, we provide the updated map computed using the approach in [7] during the robot navigation. On the other hand, in the right column of Figure A6, the maps created with the proposed approach are reported. The final maps obtained from the two approaches are shown in Figure A7. From a qualitative point of view, we can note that our old approach provides a wrong updated map compared to the ground truth one of Figure A7c. Indeed, the method wrongly changed the status of some cells belonging to the walls and correctly updated the status of some cells, highlighted in yellow, but in a wrong position, as we can see comparing Figure A6e and Figure A7. Instead, our new approach started to update the map later, but the resulting map is more precise.

Furthermore, to quantify the increase in map quality with our new approach, we applied the metrics described in Section 6.1 in order to compare the two updated maps with the reference ground truth $G_{2}$. The results are in Table A1, where a 100% score is a full correspondence of the two maps. It is worth noting that not updating the obstacles’ internal cells affects the first two metrics, but not the third, which reports more accurate results. For each metric, the percentage value for the procedure proposed in previous work and the one introduced here with the localisation check module is reported. The updated map provided by our new approach with the localisation check module has, for each metric, a higher percentage value with respect to the one built without that module when compared with the ground truth map, and this motivates the need for such a module.

**Table A1:** Map quality comparison on different metrics.

	Old $M_{2}$/$G_{2}$	New $M_{2}$/$G_{2}$
CC (%)	66.86	70.03
MS (%)	55.11	61.24
OPDF (%)	90.40	95.87

### Appendix A.4. Case of Compromised Localisation

For the simulation results, we assumed that the robot’s initial pose was known to reflect the conditions of an industrial task as closely as possible. However, aware that the robot could still get lost, we also developed a pose-updating procedure to ensure that our map-updating process can continue properly. In this section, though, we give an example of what happens when the robot gets lost and what is actually the condition (7) for which the map-updating system is suspended due to localisation errors, and the localisation system cannot recover the pose. In this example, the same worlds and maps of Section 6.3.1 are used, but this time, the filter is initialized, with an initial estimated pose in ${\tilde{X}}_{R}\left(0\right)=\left[1.8,7.5,0\right]$, with an error of around 1.5m, as we can see from Figure A8a,b. Since the localisation error is too big, the localisation system is unable to recover the robot pose, and condition (7) is verified. At this point, the map-updating process is suspended, and the robot continues to move. As we can see from the system evolution in Figure A8c,e, the robot gets lost, and the map $M_{2}\left(20\right)$ has not been updated, and hence coincides with $M_{2}\left(0\right)$, but it does not represent the world $W_{2}$. In order to make the system robust to such occurrences, a dedicated pose recovery module was developed and integrated with the map-updating process, as described next.

### Appendix A.5. Pose-Updating Algorithm in Best-Case Scenario

To assess the performance of the pose-updating algorithm module in nominal conditions, e.g., the best-case scenario, an experiment is performed where the map used by the robot to localise itself reflects the current environment. During navigation, the robot’s estimated pose is manually changed through the ROS service (see the green arrow in Figure A9b). As desired, the new pose violates condition Equation (7), as represented by the red arrows in Figure A9c, and the robot gets lost. Finally, the *pose update* module is activated, and the robot pose is correctly retrieved (see Figure A9d).

### Appendix A.6. Localisation Performance Results in the Simulated World W 4

In this appendix, the analysis of localisation uncertainty in world $W_{4}$ is reported in Figure A10, from which we draw the same conclusions reported in Section 6.4.2.

## References

[1] Chong T., Tang X., Leng C., Yogeswaran M., Ng O., Chong Y. (2015). Sensor Technologies and Simultaneous Localization and Mapping (SLAM). Procedia Comput. Sci..

[2] Dymczyk M., Gilitschenski I., Siegwart R., Stumm E. Map summarization for tractable lifelong mapping. Proceedings of the RSS Workshop.

[3] Sousa R.B., Sobreira H.M., Moreira A. (2023). A Systematic Literature Review on Long-Term Localization and Mapping for Mobile Robots. J. Field Robot..

[4] Meyer-Delius D., Hess J., Grisetti G., Burgard W. Temporary maps for robust localization in semi-static environments. Proceedings of the 2010 IEEE/RSJ International Conference on Intelligent Robots and Systems.

[5] Shaik N., Liebig T., Kirsch C., Müller H. (2017). Dynamic map update of non-static facility logistics environment with a multi-robot system. Proceedings of the KI 2017: Advances in Artificial Intelligence: 40th Annual German Conference on AI.

[6] Abrate F., Bona B., Indri M., Rosa S., Tibaldi F. Map updating in dynamic environments. Proceedings of the ISR 2010 (41st International Symposium on Robotics) and ROBOTIK 2010 (6th German Conference on Robotics).

[7] Stefanini E., Ciancolini E., Settimi A., Pallottino L. Efficient 2D LIDAR-Based Map Updating For Long-Term Operations in Dynamic Environments. Proceedings of the 2022 IEEE/RSJ International Conference on Intelligent Robots and Systems (IROS).

[8] Quigley M., Conley K., Gerkey B., Faust J., Foote T., Leibs J., Wheeler R., Ng A. (2009). ROS: An Open-Source Robot Operating System. http://robotics.stanford.edu/~ang/papers/icraoss09-ROS.pdf.

[9] Banerjee N., Lisin D., Lenser S.R., Briggs J., Baravalle R., Albanese V., Chen Y., Karimian A., Ramaswamy T., Pilotti P. (2023). Lifelong mapping in the wild: Novel strategies for ensuring map stability and accuracy over time evaluated on thousands of robots. Robot. Auton. Syst..

[10] Amigoni F., Yu W., Andre T., Holz D., Magnusson M., Matteucci M., Moon H., Yokotsuka M., Biggs G., Madhavan R. (2018). A Standard for Map Data Representation: IEEE 1873–2015 Facilitates Interoperability Between Robots. IEEE Robot. Autom. Mag..

[11] Thrun S. (2003). Robotic Mapping: A Survey. Exploring Artificial Intelligence in the New Millennium.

[12] Sodhi P., Ho B.J., Kaess M. Online and consistent occupancy grid mapping for planning in unknown environments. Proceedings of the 2019 IEEE/RSJ International Conference on Intelligent Robots and Systems (IROS).

[13] Thrun S., Burgard W., Fox D. (2005). Probabilistic Robotics.

[14] Meyer-Delius D., Beinhofer M., Burgard W. Occupancy Grid Models for Robot Mapping in Changing Environments. Proceedings of the AAAI.

[15] Baig Q., Perrollaz M., Laugier C. (2014). A Robust Motion Detection Technique for Dynamic Environment Monitoring: A Framework for Grid-Based Monitoring of the Dynamic Environment. IEEE Robot. Autom. Mag..

[16] Nuss D., Reuter S., Thom M., Yuan T., Krehl G., Maile M., Gern A., Dietmayer K. (2018). A random finite set approach for dynamic occupancy grid maps with real-time application. Int. J. Rob. Res..

[17] Huang J., Demir M., Lian T., Fujimura K. An online multi-lidar dynamic occupancy mapping method. Proceedings of the 2019 IEEE Intelligent Vehicles Symposium (IV).

[18] Llamazares A., Molinos E.J., Ocana M. (2020). Detection and Tracking of Moving Obstacles (DATMO): A Review. Robotica.

[19] Biber P., Duckett T. Dynamic Maps for Long-Term Operation of Mobile Service Robots. Proceedings of the Robotics: Science and Systems.

[20] Banerjee N., Lisin D., Briggs J., Llofriu M., Munich M.E. Lifelong mapping using adaptive local maps. Proceedings of the 2019 European Conference on Mobile Robots (ECMR).

[21] Tsamis G., Kostavelis I., Giakoumis D., Tzovaras D. Towards life-long mapping of dynamic environments using temporal persistence modeling. Proceedings of the 2020 25th International Conference on Pattern Recognition (ICPR).

[22] Wang L., Chen W., Wang J. Long-term localization with time series map prediction for mobile robots in dynamic environments. Proceedings of the 2020 IEEE/RSJ International Conference on Intelligent Robots and Systems (IROS).

[23] Sun D., Geißer F., Nebel B. Towards effective localization in dynamic environments. Proceedings of the 2016 IEEE/RSJ International Conference on Intelligent Robots and Systems (IROS).

[24] Hu X., Wang J., Chen W. Long-term Localization of Mobile Robots in Dynamic Changing Environments. Proceedings of the 2018 Chinese Automation Congress (CAC).

[25] Pitschl M.L., Pryor M.W. Obstacle Persistent Adaptive Map Maintenance for Autonomous Mobile Robots using Spatio-temporal Reasoning*. Proceedings of the 2019 IEEE 15th International Conference on Automation Science and Engineering (CASE).

[26] Lázaro M.T., Capobianco R., Grisetti G. Efficient long-term mapping in dynamic environments. Proceedings of the 2018 IEEE/RSJ International Conference on Intelligent Robots and Systems (IROS 2018).

[27] Zhao M., Guo X., Song L., Qin B., Shi X., Lee G.H., Sun G. A General Framework for Lifelong Localization and Mapping in Changing Environment. Proceedings of the2021 IEEE/RSJ International Conference on Intelligent Robots and Systems (IROS).

[28] Amanatides J., Woo A. A Fast Voxel Traversal Algorithm for Ray Tracing. Proceedings of the 8th European Computer Graphics Conference and Exhibition, Eurographics 1987.

[29] Zhang Z., Ikeuchi K. (2014). Iterative Closest Point (ICP). Computer Vision: A Reference Guide.

[30] Myronenko A., Song X. (2010). Point Set Registration: Coherent Point Drift. IEEE Trans. Pattern Anal. Mach. Intell..

[31] Grupp M. (2017). evo: Python Package for the Evaluation of Odometry and SLAM. https://github.com/MichaelGrupp/evo.

[32] Macenski S., Jambrecic I. (2021). SLAM Toolbox: SLAM for the dynamic world. J. Open Source Softw..

[33] Fox D., Burgard W., Dellaert F., Thrun S. Monte Carlo Localization: Efficient Position Estimation for Mobile Robots. Proceedings of the 16th National Conference on Artificial Intelligence (AAAI ’99).

[34] Robotics A.W.S. aws-robomaker-small-house-world. https://github.com/aws-robotics/aws-robomaker-small-house-world.

[35] Koenig N., Howard A. Design and use paradigms for Gazebo, an open-source multi-robot simulator. Proceedings of the 2004 IEEE/RSJ International Conference on Intelligent Robots and Systems (IROS) (IEEE Cat. No.04CH37566).

